# Older adults’ recognition of medical terminology in hospital noise

**DOI:** 10.1186/s41235-024-00606-1

**Published:** 2024-12-05

**Authors:** Tessa Bent, Melissa Baese-Berk, Brian Puckett, Erica Ryherd, Sydney Perry, Natalie A. Manley

**Affiliations:** 1 Department of Speech, Language and Hearing Sciences, Indiana University, Tessa Bent, 2631 E. Discovery Parkway, Bloomington, IN 47408 USA; 2 Department of Linguistics, University of Chicago, Chicago, USA; 3 Durham School of Architectural Engineering and Construction, University of Nebraska-Lincoln, Lincoln, USA; 4 Division of Geriatrics, Gerontology and Palliative Medicine, University of Nebraska Medical Center Department of Internal Medicine, Omaha, USA

**Keywords:** Older adults, Hospital noise, Intelligibility, Lexical characteristics, Speech perception

## Abstract

Word identification accuracy is modulated by many factors including linguistic characteristics of words (frequent vs. infrequent), listening environment (noisy vs. quiet), and listener-related differences (older vs. younger). Nearly, all studies investigating these factors use high-familiarity words and noise signals that are either energetic maskers (e.g., white noise) or informational maskers composed of competing talkers (e.g., multitalker babble). Here, we expand on these findings by examining younger and older listeners’ speech-in-noise perception for words varying in both frequency and familiarity within a simulated hospital noise that has important non-speech information. The method was inspired by the real-world challenges aging patients can face in understanding less familiar medical terminology used by healthcare professionals in noisy hospital environments. Word familiarity data from older and young adults were collected for 800 medically related terms. Familiarity ratings were highly correlated between the two age groups. Older adults’ transcription accuracy for sentences with medical terminology that vary in their familiarity and frequency was assessed across four listening conditions: hospital noise, speech-shaped noise, amplitude-modulated speech-shaped noise, and quiet. Listeners were less accurate in noise conditions than in a quiet condition and were more impacted by hospital noise than either speech-shaped noise. Sentences with low-familiarity and low-frequency medical words combined with hospital noise were particularly detrimental for older adults compared to younger adults. The results impact our theoretical understanding of speech perception in noise and highlight real-world consequences of older adults’ difficulties with speech-in-noise and specifically noise containing competing, non-speech information.

**Significance statement**

During hospital stays, patients receive a wealth of information from their healthcare providers, the understanding of which is critical for promoting positive health outcomes. Yet, the language used by healthcare providers and the noisy hospital environment may reduce communication success. To address how these barriers may impact communication in hospitals, we applied foundational concepts in hearing science and speech perception to investigate how medical terminology and hospital soundscapes may impact speech understanding. Specifically, we tested older and younger adults’ abilities to identify less frequent and less familiar medical terminology in simulated hospital soundscapes compared to other types of noise. The results showed that listeners had particular difficulty understanding less familiar and less frequent words in hospital noise, with greater decrements for older than younger adults. Therefore, the noise present in hospital settings may lead to misunderstandings of orally presented medical information, particularly in geriatric populations.

## Introduction

Adults who are 65 years of age and older are much more likely to receive in-patient care in a hospital compared to younger adults (Sun et al., 2018). During these hospital stays, crucial information about diagnoses, treatment plans, and discharge instructions will be provided to patients. Although it is essential for all adults to understand the medical information provided to them in these hospital settings, there are several barriers to successful communication that could hinder transfer of information and ultimately impact health outcomes. First, hospitals are noisy places (Busch-Vishniac, 2019; Busch-Vishniac et al., 2005; Darbyshire & Young, 2013; De Lima Andrade et al., 2021; Gladd & Saunders, 2011; Ryherd et al., 2011; Tainter et al., 2016). Due to the increased incidence of hearing loss and other age-related physiological and cognitive changes, older adults’ understanding of spoken information in these noisy settings may be compromised (Adel Ghahraman et al., 2020; Davis et al., 2016). Second, medical information may contain words that are unfamiliar or infrequently encountered, which can also hinder language comprehension and recall. As a first step in more deeply understanding the impact of these communication barriers in a commonly encountered situation with potentially severe real-world consequences, we investigate how hospital noise impacts older adults’ abilities to understand sentences with medically related terms that vary in their word familiarity and frequency.

### Aging and perception of speech in noise

Understanding speech in noisy conditions is more difficult than in quiet conditions for listeners across the lifespan but is particularly detrimental for older adults compared to young adults. Older adults’ difficulty understanding speech in noise frequently can be traced to the incidence of age-related hearing loss (presbycusis) (Cruickshanks et al., 2010; Humes, 1996). In the USA, the prevalence of hearing loss (unilateral or bilateral) increases from approximately 3% for adults in their 20’s to 45%, 68% and 89% for adults in their 60’s, 70’s, and 80+, respectively (Lin et al., 2011). Individuals with hearing loss may understand speech well in quiet conditions but generally need more favorable signal-to-noise ratios (i.e., louder target speech relative to the background noise) to achieve similar word recognition accuracy compared to those without hearing loss (Plomp, 1986). In addition, listeners with hearing loss (Festen & Plomp, 1990) and older listeners (Dubno et al., 2002, 2003; Gifford et al., 2007; but see Schoof & Rosen, 2014) benefit less from fluctuations in the background noise than listeners with normal hearing and young adult listeners. Fluctuating background sounds are common in most everyday noisy situations, such as when there is a talker or multiple talkers in the background. Listeners can generally take advantage of these background sound dips where the target signal is more audible to piece together the intended message (Cooke, 2006; Miller & Licklider, 1950), but older listeners may be less able to take advantage of these dips due to factors such as more difficulty recovering from forward masking effects (Festen & Plomp, 1990).

Dubno et al. (1984) demonstrated that even when hearing thresholds were matched for older (65 years and older) and younger (44 years and under) adult listeners, older listeners still showed decreased speech in noise understanding compared to younger listeners. Older adults who have hearing thresholds within normal limits (i.e., pure-tone thresholds that are lower than 25 dB at octave frequencies between 250 and 3000 Hz in the better ear) also have more difficulty with certain types of background noises. For example, younger and older listeners have been found to have similar difficulties understanding speech in white noise, speech-shaped noise, or amplitude-modulated speech-shaped noise, but older adults show worse performance than younger adults when competing background signals are single or multiple talkers. (Schoof & Rosen, 2014; Tun & Wingfield, 1999).

There are numerous underlying factors that may lead to older listeners’ difficulties with these complex listening environments. Some of their challenges may be traced to their greater susceptibility to informational masking compared with energetic masking. Energetic masking refers to listening conditions in which the masking sound has energy in the same critical frequency bands at a point in time leading to inaudible portions of the target sound (Brungart, 2001; Pollack, 1975). In contrast, informational masking conditions contain a target sound and a competing masker that are both audible, but the content of the two sound signals (e.g., two simultaneously presented speech signals) must be disentangled to extract the linguistic information in the target signal (Kidd et al., 2008; Pollack, 1975). Kidd et al. (2008) note that informational masking encapsulates many different cognitive processes including “perceptual grouping and source segregation, attention, memory, and general cognitive processing abilities” (p. 143–144). Indeed, a range of studies have suggested that older adults may have difficulty with informational masking conditions due to reduced efficiency in attentional control (Tun et al., 2002), reduced processing of temporal fine structure and use of phonetic cues to support sound source segregation (Rajan & Cainer, 2008; Schoof & Rosen, 2014), and weaker neural encoding of fundamental frequency information (Anderson et al., 2011). Furthermore, there is evidence that older adults are less able to take advantage of indexical cues (i.e., differences between individual talker’s voices) and do not receive as much benefit from a spatial release from masking (i.e., benefits accrued when the target and the masker are in different physical locations compared to conditions in which they are coming from the same physical location) (Helfer et al., 2017).

In addition to masker characteristics, age group differences for speech-in-noise tasks are especially apparent for materials that contain few contextual cues; younger adults outperform older adults in open-set single word identification and with low-context sentences (e.g., *We should have considered the juice*) but show similar performance for high-context sentences (e.g., *We drank some orange juice*) or word recognition in a closed-set task (Dubno et al., 1984; Sommers & Danielson, 1999).

These results suggest that in addition to the impact of changes in hearing sensitivity, there are cognitive changes during the aging process that can impact the ability to extract meaning from spoken messages in noisy environments. There is a link between understanding speech in noise and a range of cognitive measures including working memory, attentional switching, sustained attention, selective attention, visual cognitive-linguistic measures (e.g., spatial short term memory in which participants recalled locations of dots on a grid), and complex non-speech auditory measures (e.g., environmental sound identification) (Füllgrabe et al., 2015; Humes et al., 2013); beyond age, declines in cognitive abilities independently contribute to understanding speech in noise (Moore et al., 2014). Sensory impairments, such as hearing loss, also increase the risk of hospital delirium (George et al., 1997), which impacts cognition, alertness, and ability to pay attention, all essential factors in understanding speech.

The body of work investigating older adults’ speech perception abilities in noise strongly suggests that noisy environments, such as hospitals, may cause communication challenges, beyond those experienced by younger adults. Hospital soundscapes include specific characteristics known to disrupt speech understanding in older adults, such as fluctuating noise, as well as linguistic messages known to be challenging for this population such as low-context sentences. Furthermore, patients with hearing loss who wear hearing aids frequently leave their hearing aids at home during hospital stays for fear of losing them (Blustein et al., 2018). These factors suggest that miscommunications between older patients and healthcare providers could arise due to environmental or linguistic factors or their combination. The consequences of these communication challenges are substantial as there is evidence that higher rates of communication trouble between older adults and healthcare providers lead to increases in hospital readmissions (Chang et al., 2018).

### Aging and vocabulary

While hearing sensitivity shows a consistent decline across adulthood, other cognitive abilities continue to improve with peak performance shown at different ages. Many cognitive abilities (e.g., memory, speed, reasoning, attention) show peaks in the 20’s, 30’s, or 40’s with steady declines in later ages (Fortenbaugh et al., 2015; Hartshorne & Germine, 2015; Salthouse, 2019). However, the apex of vocabulary knowledge tends to appear much later compared to other cognitive abilities with peaks in later adulthood (50’s or 60’s). There is also evidence that older adults (those in their 70’s and 80’s) have larger vocabularies than younger adults (those in their 20’s through 40’s) (Gold et al., 1995; Salthouse, 2019) and are better at estimating their vocabulary knowledge than younger adults (Kavé & Halamish, 2015).

There are also age-related decrements in aspects of acquiring new words that may hinder adults’ speech understanding. For example, the ability to extract meaning from context for unfamiliar words decreases with age (McGinnis & Zelinski, 2000; Zelinski & Hyde, 1996). The difficulty of determining precise meaning from context appears to be greater in old-old (over 75 years of age) adults compared to young-old (65–74 years of age) or young adults (18–37 years of age) and is related to declines in generalized inferential processing (McGinnis & Zelinski, 2003). Furthermore, older adults are slower at using semantic context to predict upcoming words (Harel-Arbeli et al., 2021).

The vocabulary abilities of older adults suggest that, unlike their disadvantages for speech-in-noise perception, their verbal processing abilities may give them some advantages compared to younger adults in contexts with more complex or less frequently encountered words, such as in medical settings. Individuals, including both younger and older adults, with larger vocabularies have an advantage for understanding speech in adverse conditions (Carroll et al., 2016; Kaandorp et al., 2016; McAuliffe et al., 2013). However, their reduced abilities to extract precise meanings from context for unfamiliar words could also be a barrier for successful communication with their healthcare providers.

In this paper, we describe three experiments investigating knowledge and perception of medically related terminology. The first experiment assesses older adults’ familiarity with a large set of medically related words and compares their scores to those of young adults. The second experiment employs a subset of these words in a speech-in-noise intelligibility task in which the listening environment was also manipulated to compare quiet, hospital noise, and speech-shaped noise conditions using a between-subjects design. This experiment is a conceptual replication of a study with young adults (Bent et al., 2022). The third experiment employs a mixed design where noise type is a within-subjects variable and tests new cohorts of young and older adults. In addition to having the noise type as a within-subjects manipulation for this experiment, a fourth listening condition is included in which a speech-shaped noise was amplitude-modulated based on the amplitude envelope of the hospital noise. For all experiments, participants were recruited and tested online. Remote testing has been shown to be an effective methodology for testing older adults’ perception of speech in noise (e.g., Brown et al., 2021), with results showing no significant differences for speech-in-noise transcription accuracy performance for remote vs. in-lab tested listeners with signal-to-noise ratios similar to the one employed here (Shen & Wu, 2022).

### Openness and transparency statement

For all three experiments reported below, we report how we determined our sample sizes, all data exclusions, all manipulations, and all measures in the study. All data, analysis code, and research materials are available on our Open Science Framework page: https://osf.io/rvz2f/. Data were analyzed using R, version 4.1.2 (R Core Team, 2021), and the package *ggplot*, version 3.3.5 (Wickham, 2016). All procedures were approved by the Institutional Review Board at Indiana University (Protocol # 2004206295). This study was not preregistered.

## Experiment 1: familiarity ratings for medically related terms

Although prior studies have shown high agreement between older and younger adults in their familiarity ratings of pictures (Yoon et al., 2004) and for subjective frequency ratings (Balota et al., 2001), it is possible that there could be age or cohort effects for words related to healthcare. There is evidence that performance in visual word recognition is better predicted when word ratings (e.g., subjective frequency or age of acquisition estimates) are derived from participants of the same age cohort (Dorot & Mathey, 2010)**.** Thus, the familiarity estimates gathered in this experiment may provide more accurate modeling of the intelligibility data for the older adults in Experiments 2 and 3 rather than using familiarity ratings from a cohort of young adults.

### Method

#### Participants

Fifty monolingual American English speakers between the ages of 60 and 75 (mean = 65) completed the familiarity rating task. Participants included 37 women and 13 men. One participant identified as Hispanic or Latinx and the others did not. The race of the participants included white (*n* = 43), Black or African American (*n* = 2), American Indian or Alaska native and white (*n* = 2), and other (*n* = 2). One participant preferred not to indicate their race. Four participants indicated that they had some degree of hearing loss. Participants rated their exposure to medical professions as minimal (~ once per year or less; *n* = 14), low moderate (~ a few times per year; *n* = 29), or moderate (~ once per month; *n* = 7). Four additional participants were tested, but their data were not included due to daily interactions with medical professionals (*n* = 2), fewer than 80% correct on the attention checks (*n* = 1), or fewer than 90% of reliability checks correct (*n* = 1). For this final category, we extracted the ratings for 20 words that should be highly familiar to all participants (e.g., *help*, *life*) and were rated as highly familiar (all “7” ratings on a scale of 1–7) in the HoosierFAM dataset (Nusbaum et al., 1984), which includes ratings from 600 participants. If participants did not give a rating of “7” to at least 90% of these words, they were excluded. Our sample size was calculated so that each stimulus item would receive ratings from at least 20 participants.

The older adult data were compared to previously collected younger adult data (Perry et al., 2021). The younger adult data included a sample of 41 monolingual American English speakers between the ages of 18–35. Exclusion criteria were the same ones used with the older adult sample described in the previous paragraph (e.g., daily interaction with medical professionals, attention checks, low ratings for highly familiar words).

#### Stimuli

Eight hundred words from Merriam-Webster’s online medical dictionary (*Medical Dictionary by Merriam-Webster*, n.d.) were included. These words were selected to elicit a range of familiarity ratings and were the same words assessed for younger adults in Perry et al. (2021).

#### Procedure

Participants were recruited online via Prolific (https://prolific.co/) and tested in 2021. The experiment would appear to potential participants if they met all inclusion criteria, including the target age range (60–85), living in the USA, American citizen, and native monolingual speaker of English. If they decided to complete the study, they were redirected to Qualtrics, where they completed the consent process, background questionnaire, and word familiarity rating task.

For the rating task, each participant was orthographically presented with a randomly selected subset of 400 words. Their task was to rate each word on a scale of 1–7 (Table 1) (Nusbaum et al., 1984). The words were divided into sets of 20 that included 18–19 words and 1–2 attention checks. For the attention check, participants were required to click on a specific number on the rating scale (e.g., “select rating 2”).

**Table 1:** Descriptions of the points on the familiarity rating scale

Rating	Description
1	You have never seen or heard this word before
2	You think that you might have seen or heard this word before
3	You are pretty sure that you have seen or heard the word before, but you are not positive
4	You recognize the word as one you have seen or heard before, but you only have a vague idea of its meaning
5	You are certain that you have seen the word, but you only have a vague idea of its meaning
6	You think you know the meaning of the word but are not certain that the meaning you know is correct
7	You recognize the word and are confident you know the meaning of the word

### Results

**Fig. 1:**
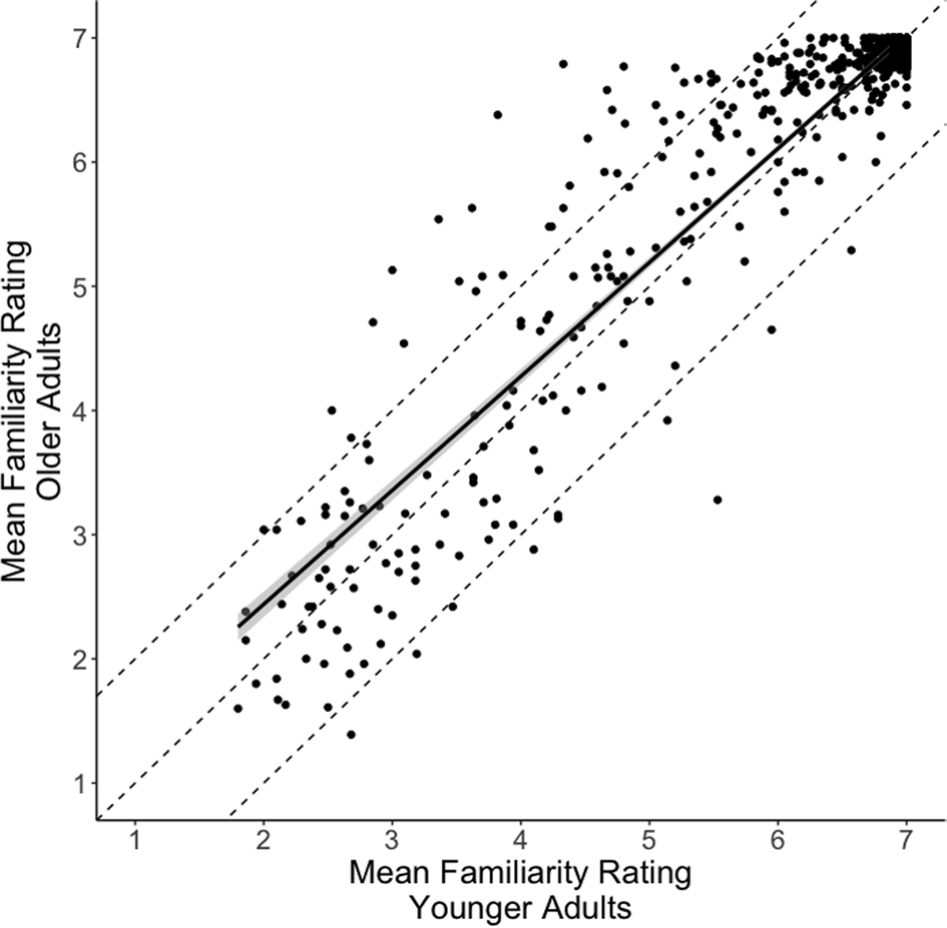
Correlation between familiarity ratings for older and younger adults. Each point represents average ratings from the two listener groups for one word. The black line is the regression fit line and the grayed in area is the 95% confidence region. The middle dashed line is where points would fall if both groups provided the same rating of a word. The area inside the outer two dashed lines represents all the items for which older and younger adults had mean scores within one point on the rating scale. Points outside of this area represent ratings differences that were greater than one point between the two age groups

Ratings for each word were averaged across the participants. Each word received ratings from an average of 25 participants with a minimum of 23 ratings. These ratings were compared to ratings previously gathered from the group of young adult participants (Perry et al., 2021). The ratings between the two age groups were highly correlated, *r *(798) = 0.941, *p* < 0.001^1^ (Fig. 1). Furthermore, the average ratings across all the words were similar for the two groups (younger adults = 6.26; older adults = 6.35). Only 6% of words had scores that differed by more than 1 rating point between the two groups. There were 10 words where the young adult ratings were more than 1 point higher than the older adult ratings and 36 words where the older adult ratings were more than 1 point higher than the younger adult ratings. Many of the words (*n* = 558) received high mean ratings (≥ 6.5) from both age groups.

### Discussion

Most of the medically related words included in our sample received very similar familiarity ratings across the two age groups suggesting that using word familiarity norms collected from young adults may be appropriate to use with older adult populations, even for medical terminology. We expected that the older adult ratings would be substantially higher than the younger adults because there is strong evidence that vocabulary increases with age (e.g., Verhaeghen, 2003). However, the ratings were very similar across the two age groups with no clear patterns of higher ratings for the older adults compared to the younger adults’ ratings. Additionally, it seemed likely that some of the words included here would have elicited larger differences between the two age groups due to the likelihood that older adults would have more experience with medical terminology due to both their greater life experiences generally and likelihood of medical problems in older age. Again, there was not a general pattern of higher ratings from the older adults even for a subset of words. The words that older adults gave higher ratings to compared to the younger adults were primarily words in which the older adults indicated that they were highly familiar, but the younger adults rated them more in the middle of the scale (e.g., *obstetrician* 6.8 vs. 4.3; *geriatric* 6.8 vs. 4.8; *halitosis* 6.6 vs. 4.7; *urea* 6.4 vs. 3.8). In contrast, the words in which the younger adults had substantially higher ratings tended to be rated by both groups more toward the middle of the scale (e.g., *phenotype* 5.5 vs. 3.3; *exothermic* 5.1 vs. 3.9; *frenulum* 4.1 vs. 2.9; *septal* 4.3 vs. 3.1). The words in which the older adults provided higher familiarity ratings, therefore, may be more driven by their real-world experiences and following certainty about the meanings of the words. For example, it is not surprising that older adults would have greater familiarity with the word “geriatric” than young adults. In contrast, the young adults may have been more generous with their ratings for words that fell more in the middle of the scale as there are not clear reasons why younger people would have greater knowledge of words such as *frenulum* or *exothermic*. However, these ratings are not based on assessment of accurate knowledge of the words, but on the participants’ own evaluation of how familiar they are with specific terms. There may be age differences in strategies or biases for rating familiarity in addition to differences in word knowledge. Previous work suggests that older adults show a close alignment between their actual word knowledge and judgments of their knowledge while young adults tend to underestimate their word knowledge (Kavé & Halamish, 2015). Therefore, it is possible that the older adult ratings may be more closely aligned with actual knowledge and therefore more predictive of speech-in-noise intelligibility. However, the substantial collinearity between the ratings for the two groups suggests that word ratings from either age group would be sufficient for modeling the impacts of word familiarity.

## Experiment 2: conceptual replication of Bent et al. (2022) with older adults

Most of the studies investigating how different masker types impact listeners from varied age cohorts compare non-speech maskers (e.g., speech-shaped noise or amplitude fluctuating noise) to speech maskers (i.e., maskers with different numbers of competing talkers), noting that older listeners have particular difficulty with maskers that contain intelligible speech. However, maskers can contain information that is not speech, but still may be salient to listeners. Here, we compare older listeners’ performance for a masker that results in energetic masking only with one that also includes informational masking, including clearly identifiable sounds, but little to no identifiable semantic information.

For this informational masking signal, we use a particularly important and ecologically valid noise type: hospital noise. Only two studies to date have investigated how hospital noise influences listeners’ word identification (Bent et al., 2022; Pope et al., 2013). In both studies, when sentences were presented in hospital noise, listeners were less accurate at identifying the words compared to quiet conditions. However, the impact of aging could not be determined in either study. Bent et al. (2022) only included young adult listeners. Although Pope et al. (2013) included listeners from a wide age range from late 20’s to late 70’s with an average of 54, the authors did not include age as a factor in their analysis and therefore it is not possible to know whether the older adults had more difficulty with the tasks when they were presented in hospital noise compared to the younger listeners. Furthermore, the sentences included in the Pope et al. study were standard speech perception sentences that manipulated the availability of context but did not contain medical terminology (e.g., high context: *Stir your coffee with a spoon* vs. low context: *Bob could have known about the spoon*).

Here we address these gaps by testing older adults’ perception of sentences that include medical terminology within three listening conditions—quiet, hospital noise, and speech-shaped noise—using the same design and materials as in Bent et al. (2022) to allow for a conceptual comparison with young adult performance.

### Method

#### Participants

Seventy-nine monolingual American English-speaking adults between the ages of 60 and 81 (average = 66) participated. Participants included 45 women and 34 men. One participant identified as Hispanic or Latinx and the others identified as not Hispanic or Latinx. Seventy-three participants identified as white and six participants as Black or African American. Five participants indicated some hearing issues including the following: “airplane ear”, tinnitus, mild hearing loss in the high frequencies, age-related hearing loss, and moderate hearing loss and use of hearing aids. All participants indicated that they did not have any diagnosed cognitive impairment. Participants rated their exposure to medical professions as minimal (~ once per year or less; *n* = 16), low moderate (~ a few times per year; *n* = 50) or moderate (~ once per month; *n* = 13). They rated the level of background noise in their environment as 2.2 on a 1 (very quiet) to 10 (very loud scale) (range = 1–6). An additional eight participants were tested but their data were not included due to bilingual language status (*n* = 3), cognitive impairment (*n* = 1), frequent exposure to medical professionals (*n* = 2), or a rating of 8 or 9 on the 10-point scale of background noise in the environment (*n* = 2). There were also 10 participants who failed the headphone check and therefore did not complete the background questionnaire or intelligibility experiment. We aimed to have 75 participants with usable data included in our study (roughly 25 per noise condition described below), but included all participants with usable data who completed the experiment during our online recruitment period on Prolific.

#### Stimuli

The stimuli were 160 sentences with three keywords each taken from the corpus described in Perry et al. (2021). Sentences were 4–9 words in length (average 6.9 words). The sentences had quantified familiarity, frequency, and predictability profiles. Three of the sentence types included medical terminology and were divided into three subtypes with specific word familiarity and frequency characteristics including 40 each with high-familiarity and high-frequency words, high-familiarity and low-frequency words, and low-familiarity and low-frequency words. Frequency categorization was determined by Zipf scores from SUBTLEX-US, a corpus of 51 million words taken from American subtitles (Brysbaert & New, 2009; van Heuven et al., 2014). Zipf scores vary from 1 to 7.

Determination of low or high familiarity for the initial corpus was based on the ratings from young adults (as described above) with scores ranging from 1 to 7. Sentence categorization was determined by the average familiarity and frequency scores for the three keywords in the sentence. Sentences in the high-frequency category had average frequency scores between 4.3 and 5.3 and sentences in the low-frequency categories had average scores between 1.7 and 3.99. Sentences in the high-familiarity categories had scores between 6.7 and 7.0 and the low-familiarity sentences ranged from 3.6 to 5.5. There was a fourth set of 40 sentences that were adapted from standardized speech perception materials (i.e., the Hearing in Noise Test; Nilsson et al., 1994). These sentences also included keywords that were high in familiarity and frequency but did not include medical terminology.

Predictability was quantified in a cloze test in which participants were presented orthographically with a sentence with one keyword missing and had to guess the missing word (e.g., It is a safe and ____ process). Predictability scores represent the percentage of correctly guessed words averaged across the three keywords in each sentence. Standard sentences had the highest predictability (19.6%) followed by high-familiarity/high-frequency (7.0%) and high-familiarity/low-frequency (7.0%) with the lowest scores for the low-familiarity/low-frequency sentences (1.7%).

Four monolingual American English speakers (two male and two female) were recorded reading the sentences. Speakers’ dialects were either Midland (*n* = 2) or North (*n* = 2) and were between the ages of 18–29 (average = 22.5). Recordings were made in a sound-attenuated booth using a Marantz PDM670 digital recorder and a Shure Dynamic WH20XLR headset microphone. Sentences were equated for root-mean-square (RMS) amplitude.

In the experiment, sentences were presented in one of three conditions: quiet, speech-shaped noise, or hospital noise. The speech-shaped noise was created by taking the long-term average spectra of a set of sentences and using it to filter a white noise. The hospital noise was synthesized in previous work (Messingher, 2013), although it was compressed in Audacity to remove extreme peaks. This noise includes sounds frequently encountered in a hospital setting including HVAC noise, footfalls, squeaking, telephones ringing, laughter, and alarm sounds. Voices were also included, although most speech was not intelligible. The amount of intelligible speech was quantified by three research assistants. Each one listened to the entire 1780 s noise file. They annotated the sections that had intelligible speech using a textgrid in Praat. The average amount annotated as intelligible speech was 9.7% (range 7.6–11.3%). Only 6.5% of the noise file had speech where all three annotators agreed on the content of the speech. The lack of full interrater agreement was a consequence of the indistinct and noisy nature of the recording. The characteristics of the hospital noise were also designed to match the spectral content, temporal fluctuations, and other characteristics typical of real-world hospital settings. Additional details on the noise stimuli can be found in Bent et al. (2022).

**Fig. 2:**
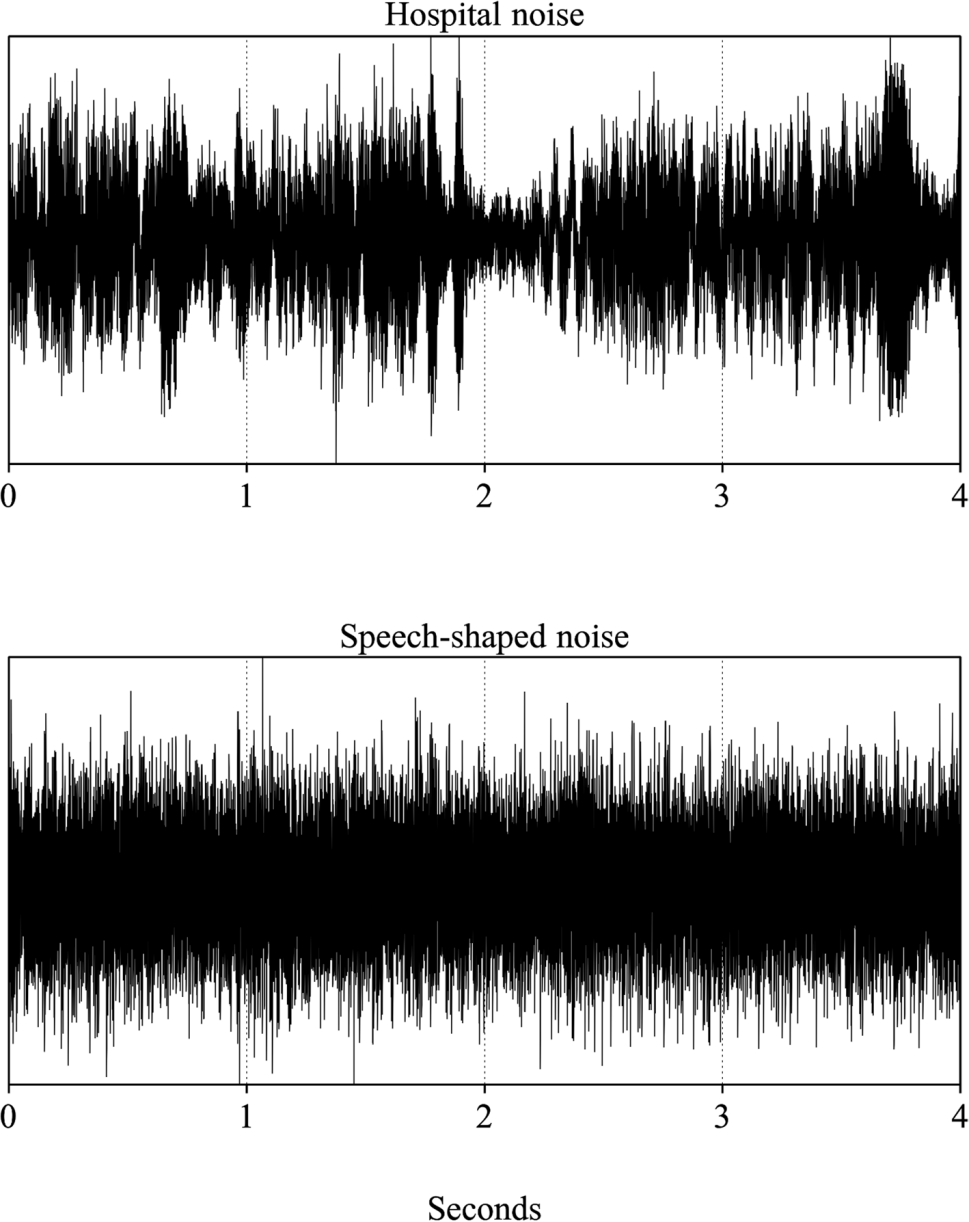
Examples of the two noises used for the intelligibility experiment. Each is a four second extract from the longer noise file. The hospital noise is shown in the top panel and the speech-shaped noise in the bottom panel

The two noise conditions were presented at a signal-to-noise ratio of − 1 dB. This SNR was selected to match the ratio used in our previous study (Bent et al., 2022) to allow conceptual comparisons to a group of young adult listeners. Each sentence was embedded in a random selection of noise (Fig. 2) that was 1 s longer than the sentence using a custom-designed script written in Python.

### Procedure

Participants were recruited through Prolific (https://www.prolific.co/) and tested in 2021. If they met the inclusion criteria (i.e., between the ages of 60–85, currently living in the USA, American citizen, and monolingual English speaker), the study would appear as available for participation. Participants who had participated in the familiarity rating task were excluded from participation. If they decided to participate in the study, they were directed to a Qualtrics survey that included the study information sheet, a headphone screening, and a background questionnaire. The headphone screening was adapted from Woods et al. (2017) and was included to ensure that participants were using headphones. Participants were provided with three opportunities to pass the screening. If they did not pass, they could not continue with the study. After the headphone screening, the background questionnaire was administered. In addition to questions about demographics, current environment, and information about interactions with medical professionals, we included the questions from the Speech Spatial and Qualities of Hearing Scale (15iSSQ) (Moulin et al., 2019). The 15iSSQ is a shortened version of the Speech Spatial and Qualities of Hearing Scale (SSQ) (Gatehouse & Noble, 2004). Both questionnaires are self-report measures of hearing ability with questions that address three aspects of hearing: Speech (e.g., questions about the difficulty in following conversation in various noisy environments), Spatial (e.g., questions about locating the position of sounds in the environment), and Qualities (e.g., distinguishing different sounds or the clarity of sounds). The scores on the 15iSSQ are significantly related to better ear pure-tone average (PTA), as measured via an audiogram (Moulin et al., 2019).

At the end of the Qualtrics questionnaire, participants were redirected to Pavlovia (https://pavlovia.org/), the online platform for PsychoPy (Peirce et al., 2019), to complete the intelligibility task. Participants used their own computers and headphones for all tasks.

For the experimental task, participants were presented with sentences and had to type in what they heard. Each participant was assigned to one of three listening conditions: quiet (*n* = 25), speech-shaped noise (*n* = 29), or hospital noise (*n* = 25). Participants were presented with all 160 sentences in randomized order. Each of the four talkers contributed 40 sentences with sentence-talker pairing counterbalanced across participants. Prior to the presentation of the experimental trials, participants were presented with four practice trials produced by a speaker who was not included in the experimental trials. Participants were not provided with any feedback and could hear each sentence only once. The task was self-paced except participants were required to take a break of at least 10 s each after every 54 trials (two required breaks total). A trial counter was shown at the top left of the screen so that participants could track their progress throughout the experiment.

### Results

Responses were scored for keyword accuracy (three keywords per sentence) in a binary fashion (i.e., correct or incorrect). Before scoring, two research assistants (author SP and one other researcher) completed a spell check on the typed responses. Obvious typos (e.g., “reconstrct” for “reconstruct”), misspellings (e.g., “esophogaus” for “esophagus”) and homophones (e.g., “pane” for “pain”) as well as phrases with added or missing spaces (e.g., “ear drum” for “eardrum” or “ultra sound” for “ultrasound”, “fluvirus” for “flu virus”) were corrected. Because sentences in the low-familiarity, low-frequency condition were by definition not highly familiar, misspellings were allowed as long as they could be pronounced as the target word (e.g., “abberation” for “aberration”, “rinitus” for “rhinitis”, “disfunction” for “dysfunction”). After the two researchers completed their spell checks, the responses were scored for accuracy. These scores were then compared and any discrepancies were resolved in consultation with a third rater (author TB). A strict scoring criterion was employed in which words with added or deleted morphemes were counted as incorrect.

These scores were then fit to a logistic mixed-effects model. We contrast-coded listening condition, so that we could directly compare the two noise conditions to the quiet condition, and then compare performance in speech-shaped noise to hospital noise. The three sentence categories—high familiarity and high frequency (HH), high familiarity and low frequency (HL), and low familiarity and low frequency (LL)—were compared such that HH served as a baseline and the other two categories were compared to it. As our previous work demonstrated that perception of the standard sentences (i.e., non-medical high-familiarity, high-frequency sentences) does not differ substantially from the HH sentences (Bent et al., 2022), we chose to only investigate the medical stimuli here. We investigated responses to the speech spatial and qualities of hearing scale (15iSSQ), but these did not improve model fit and thus are not discussed further. Participant age also did not improve model fit and is also not discussed further here. Our final model included listening condition, sentence category, and the interactions between these two effects. Random effects included random intercepts for item and for participant, which was the maximal random effect structure specified by model comparisons. Effect sizes are interpreted from the beta estimates (see Baguley, 2009 for a description of why these effect sizes are preferred to standardized effect sizes). The specifications of the final model can be seen in Table 2.

**Table 2:** Summary of logistic mixed model for Experiment 2

Predictors	Accuracy				
	Estimate	Odds ratio	Standard error	*z*	*p*
(Intercept)	1.82	6.157803	0.15	12.07	**<0 001**
Quiet versus noise conditions	− 4.53	0.010828	0.31	− 14.88	**<0.001**
Speech-shaped noise versus hospital noise	0.68	1.967355	0.23	2.97	**0.003**
HH versus HL sentence category	− 0.72	0.4880878	0.17	− 4.31	**< 0.001**
HH versus LL sentence category	− 2.49	0.0822074	0.17	− 15.06	**< 0.001**
Quiet versus noise conditions for HH versus HL sentence category	0.93	2.535498	0.19	5.04	**< 0.001**
Speech-shaped noise versus hospital noise for HH versus HL sentence category	0.09	1.093857	0.08	1.11	0.269
Quiet versus noise conditions for HH versus LL sentence category	2.51	12.344	0.17	14.71	**< 0.001**
Speech-shaped noise vs hospital noise for HH versus LL sentence category	− 0.29	0.7509021	0.09	− 3.36	**0.001**

Significant effects are shown in bold

We first investigated overall performance across noise types. It is clear from Fig. 4 that participants performed less well in the two noise conditions than in the quiet condition, and that participants appear to perform less well in the hospital noise than speech-shaped noise. Indeed, this observation is supported by the statistical analysis, which shows that word recognition accuracy in the noise conditions is significantly worse than in the quiet condition (*z* = − 14.851, *p* < 0.001), and that performance in the hospital noise condition is significantly worse than in the speech-shaped noise condition (z = 2.968, *p* = 0.003).

**Fig. 3:**
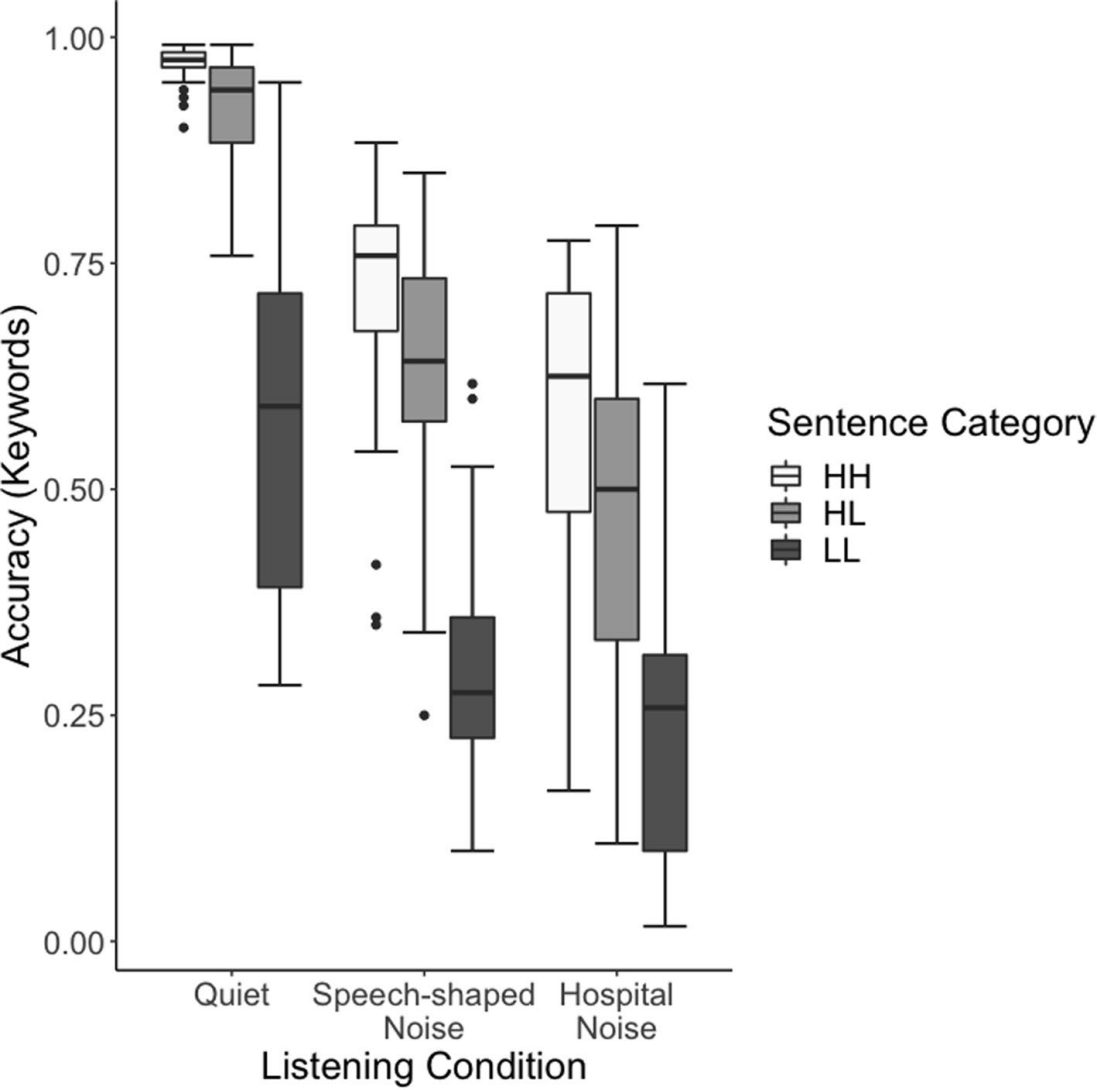
Accuracy across three listening conditions and three sentence categories

We next investigated how accuracy changes across sentence category and listening condition, as shown in Fig. 3. As expected, with regard to sentence category, participants performed less well on the high-familiarity, low-frequency stimuli (*z* = − 4.307, *p* < 0.001) and on the low-familiarity, low-frequency stimuli (*z* = − 15.066, *p* < 0.001) than on the high-familiarity, high-frequency stimuli. Further, there were significant interactions between stimulus type and listening condition, as demonstrated in Fig. 3. The listeners in our sample have an especially difficult time with sentences containing low-familiarity and low-frequency items, as compared to the other two categories. It is important to note here that frequency and familiarity are often strongly correlated; however, significant previous work has demonstrated that these two factors can have separable effects on processing (e.g., Connine et al., 1990). The statistical analysis suggests that the specific challenge of low-familiarity, low-frequency stimuli is not equal across listening conditions. When comparing the high-familiarity, high-frequency items to the high-familiarity, low-frequency items across the quiet and noise conditions, we see a significant result (*z* = 5.038, *p* < 0.001), suggesting that noise is more detrimental in cases of lower frequency. Similarly, we see the increased difficulty for low-familiarity, low-frequency words when comparing the quiet condition to the two noise conditions (*z* = 14.691, *p* < 0.001). When comparing the high-familiarity, high-frequency items to the low-familiarity, low-frequency items, in the speech-shaped vs. hospital noise, the interaction is significant (*z* = − 3.356, *p* < 0.001); however, this interaction is not significant when comparing the high-familiarity, high-frequency stimuli and the high-familiarity, low-frequency stimuli (*z* = 1.105, *p* = 0.26). That is, although low-familiarity, low-frequency items are more difficult than the high-familiarity, high-frequency items in both noise conditions, there is a larger difference between the two item types in speech-shaped noise than in hospital noise. The effect of frequency for the high-familiarity items (i.e., high-familiarity, high-frequency and high-familiarity, low-frequency items) does not differ across noise conditions. It is important to note that this interaction may be, in part, due to the apparent floor effect for the low-familiarity, low-frequency items in the hospital noise listening condition.

We also investigated whether the two familiarity measures (i.e., ratings from younger and older adults) may impact our results differently. Our analyses suggest that, because these two measures are so strongly correlated, they do not differentially impact performance. For example, inclusion of familiarity ratings for older adults results in a model with an Akaike Information Criterion (AIC) of 26,928. Inclusion of familiarity ratings for younger adults results in a model with an AIC of 26,927.5. Bayesian Information Criterion (BIC) is similarly not impacted by which ratings are included (26,994 for older adults’ familiarity vs. 26,993.5). The differences between these two models are not significantly different.

Taken together, these results suggest that older adult listeners demonstrate worse speech perception performance in noise than quiet, especially in hospital noise compared to speech-shaped noise. Further, they demonstrate reduced performance on sentences containing low-familiarity, low-frequency words compared to high-familiarity, high-frequency words or high-familiarity, low-frequency words. These two listening challenges also interact, impacting older adult listeners’ performance on sentence recognition tasks. Specifically, it appears that low-frequency items impact performance in both noise conditions to a similar degree; however, the lack of difference between the two noise conditions may, rather, be a function of the apparent floor effect for low-familiarity, low-frequency items in hospital noise.

### Discussion

The results of Experiment 2 highlight two challenges that older adults may face during interactions with healthcare providers in hospitals: noisy environments and words with low frequency and/or familiarity. In terms of the first challenge, the older adults in this study showed general difficulty understanding speech in noise compared to the quiet condition. Although listeners of all ages tend to have more difficulty understanding speech in noise compared to quiet conditions, older adults tend to have greater difficulty understanding speech in noisy conditions than young adults (Dubno et al., 1984). The reasons for these decrements frequently can be traced to hearing loss and the cognitive changes associated with the aging process (Füllgrabe et al., 2015; Moore et al., 2014; Plomp, 1986; Tun et al., 2002).

Beyond general difficulties understanding speech in noise, the older adults showed lower word recognition accuracy when sentences were presented in hospital noise compared to speech-shaped noise. These results contrast with findings from Bent et al. (2022) in which young adults showed similar performance for the same materials in the same speech-shaped noise and hospital noise conditions. There are several reasons why older listeners in particular may have difficulty understanding speech when presented in hospital noise compared to speech-shaped noise. First, the hospital noise varied in amplitude over time compared to the speech-shaped noise that was more consistent in the amplitude domain. Prior work has demonstrated that older adults show reductions in the ability to take advantage of amplitude dips in the masking signal (Dubno et al., 2002; Helfer & Freyman, 2008) due to age-related declines in temporal auditory processing (Stuart & Phillips, 1996). Therefore, in a setting like a hospital in which sounds substantially vary in loudness over time, including some with very loud amplitudes (e.g., alarm sounds), older adults may show difficulty understanding speech from healthcare providers, even if there are intermittent periods in which the signal-to-noise ratio is more favorable. Younger listeners, on the other hand, may better use the glimpses in which the target speech is more audible to accurately piece together the intended message.

A second potential cause of older listeners’ difficulty with hospital noise is problems with stream segregation, that is separating the target speech from other sounds in the environment. Stream segregation abilities of young and older adults are similar for speech-shaped noise, but older adults show more challenges in stream segregation with multitalker babble (Ben-David et al., 2012). The hospital noise employed here shows some overlap in characteristics with multitalker babble, including the amplitude variation described above, but includes a wide range of environmental sounds rather than only speech. Furthermore, the voices included in our hospital noise were nearly all unintelligible speech whereas maskers with speech can include intelligible speech, depending on the number of talkers, thus leading to greater linguistic, informational masking than present in our masker. A different experimental paradigm would need to be employed to determine how much stream segregation contributed to older adults’ challenges with the hospital noise condition, which would be a fruitful direction for future work.

A final contributor to the decrements in performance for hospital noise could be older adults’ reduced inhibitory control (Christ et al., 2001; Lustig et al., 2001). If older adults were less able to ignore the irrelevant hospital noise sounds, they would have fewer processing resources to allocate for interpreting the target speech. Reduced efficiency in attentional control has also been put further as an explanation for older adults’ difficulties in recalling speech when there is competing speech in the background (Tun et al., 2002). Since the speech-shaped noise used here was relatively constant over time and did not include any meaningful sounds, it may have been easier to ignore and captured less of the listeners’ attention. In contrast, there were clearly identifiable environmental sounds (e.g., alarm sounds, footsteps, phone ringing) and human non-speech vocalizations (e.g., laughter, coughing) present in the hospital noise. Thus, with meaningful, attention capturing sounds that changed over time, it may have been difficult for older adults to inhibit them. Furthermore, some of these sounds, such as alarms and coughing, may trigger the neural pathways that process feelings of fear and safety, attracting substantial attention. However, most of these meaningful sounds were not linguistic. Although the hospital noise used here included voices, the speech tended to be unintelligible. Thus, our hospital noise shares more in common with other real-world maskers, such as traffic noise, classroom noise, or office noise (Bell & Buchner, 2007; Klatte et al., 2010; Shukla et al., 2018; Wong et al., 2012), than the more commonly employed speech maskers, such as multitalker babble, which only includes speech content rather than a mixture of environmental sounds with some speech.

## Experiment 3: younger and older adults’ perception of medically related sentences across listening conditions

The results of Experiment 2 suggest that hospital noise may lead to more word recognition challenges for older adults than speech-shaped noise whereas younger adults in Bent et al. (2022) showed no statistically significant difference between word recognition accuracy scores in the two noise conditions. However, both of these studies used a methodology in which noise condition was a between-subjects variable. Therefore, individual differences across listeners assigned to each condition may have influenced the results. Additionally, there are several reasons why hospital noise may lead to greater difficulty for older adults, which the design used in Experiment 2 and in Bent et al. (2022) could not disentangle. Two of the reasons older adults may have more difficulty in hospital noise compared to young adults is their less efficient use of listening in the dips (Dubno et al., 2002; Festen & Plomp, 1990) and their reduced inhibitory control (Christ et al., 2001; Lustig et al., 2001). Experiment 3 aims to address these gaps by employing a mixed design where noise type is a within-subjects variable and tests new groups of younger and older adults. Additionally, we add a new noise condition: a fluctuating masker *without* characteristics of the specific noise sources heard in hospital noise. This novel masker contains fluctuating speech-shaped noise, modulated in amplitude using the hospital noise envelope. Incorporating this additional noise condition will allow for the determination of how the fluctuations of the hospital noise contribute to possible differences across age groups without the influence of informational masking. In this experiment, we also investigate how long-term exposure to medical settings may provide perceptual advantages when listening to medically related speech in hospital noise.

### Method

#### Participants

Participants were 177 monolingual, American English-speaking adults including young adults between the ages of 18 to 35 (*n* = 87; average = 29.5 years; 33 women, 53 men, 1 nonbinary) and older adults between the ages of 60 to 79 (*n* = 90; average = 66.2 years; 55 women, 34 men, 1 non-response). Two young adults and one older adult identified as Hispanic / Latinx, one older adult preferred not to respond; the remaining participants identified as not Hispanic or Latinx. Participants identified as American Indian or Alaska native (younger *n* = 1, older *n* = 1), Asian American (younger *n* = 2; older *n* = 1), Black or African American (younger *n* = 12; older *n* = 3), white American (younger *n* = 71; older = 84), other (older *n* = 1), or prefer not to say (older *n* = 1). Highest education level achieved included some high school (younger *n* = 3), high school diploma (younger *n* = 21; older *n* = 9), some college (younger *n* = 15; older *n* = 23), associates degree (younger *n* = 6; older *n* = 9), bachelor’s degree (younger *n* = 39; older = 32), master’s degree (younger *n* = 1; older *n* = 14), and doctoral degree (younger *n* = 1; older *n* = 4).

Inclusion criteria were the same as in Experiment 2. An additional 29 participants were tested but excluded from analysis due to not falling within the specified age ranges (*n* = 1), bilingual or multilingual status (*n* = 4), frequent exposure to medical professionals (*n* = 6), cognitive impairment (*n* = 4), a rating of 8 on the 10-point scale of background noise in their current environment (*n* = 1), low-effort responses (*n* = 7), or multiple exclusion criteria (*n* = 6). Low-effort responses were defined as 20 or more trials with no response and accuracy of less than 20% for the medically related sentences. Following the procedure described in Experiment 2, we aimed to have 75 participants with usable data for both older and younger adults, but included all participants with usable data who completed the experiment during our online recruitment period on Prolific.

In addition to questions about participants’ language backgrounds and education levels, participants completed the speech spatial and qualities of hearing scale (15iSSQ) questionnaire (Moulin et al., 2019). We also collected information about participants’ healthcare experience and created a composite measure, including the frequency of interaction with healthcare providers, time spent in hospitals in the past 12 months, and time spent in non-hospital healthcare facilities in the past 12 months. Each response was converted to a scale of 1–10 and then combined for a possible score of 30.

#### Stimuli

The target stimuli used in the experiment were the same 160 sentences as used in Experiment 2. Only one speaker was used for this experiment: a female, monolingual American English speaker. Sentences were presented in four listening conditions. Three of the conditions were the same as in Experiment 2: quiet, hospital noise and speech-shaped noise. A fourth listening condition was added: a speech-shaped noise modulated by the hospital noise amplitude envelope. This noise condition had the sound level fluctuations matching the hospital noise, but information from the original hospital noise signal was removed and replaced with speech-shaped noise. As in Experiment 2, the sentences were mixed with a random portion of the noise file that was one second longer than the sentence with an SNR of − 1 dB.

#### Procedure

Testing procedures followed those in Experiment 2 except that the design included listening condition as a within-participants variable. The data were collected in 2023. Thus, all participants were presented with 40 sentences of each familiarity/frequency type; within each familiarity/frequency type participants, 10 sentences were presented for each listening condition. The combination of specific sentences with specific noise types was counterbalanced across listeners. Sentences were presented in a randomized order; there was no blocking for sentence type or noise type. Participants were presented with four practice trials prior to beginning the experimental task, one for each listening condition. Similar to Experiment 2, participants were required to take at least three 10-s breaks during the experiment.

### Analysis

To assess the accuracy between participants’ transcribed responses and the target sentences, a fuzzy string matching metric—token sort ratio (TSR) from Bosker (2021)—was calculated for each sentence. The TSR is a measure of similarity between two input strings (response and target) and returns a percentage of agreement between the two. A TSR of 0 indicates there is no match between the target and response strings, and a TSR of 100 represents a perfect match. As an example, for a response of “It is agravation the nasal passage” for the target sentence “it is aggravating the nasal cartilage,” the TSR score is 70. In a keyword accuracy score approach for this same scenario, the accuracy would be 33% (for keywords *aggravating*, *nasal*, and *cartilage*). We employed the keyword scoring approach in Experiment 2 because it was a conceptual replication of Bent et al. (2022) who used that scoring method. We decided to shift our scoring method for this experiment because the TSR scores are highly correlated with keyword accuracy scores, but remove human decision making and potential biases when correcting for misspellings. Given the less familiar terminology employed, the number of judgment calls required may impact results more than typical word recognition studies. Therefore, we believe TSR scores are appropriate for comparing the accuracy of participants’ responses.

The TSR scores were fitted to a linear mixed-effects model. Listening condition was contrast-coded such that the three noise conditions were compared to the quiet condition, hospital noise was compared to the two speech-shaped noise conditions, and the AM speech-shaped noise was compared to the standard speech-shaped noise. Previous work has found that standardized speech sentences are not significantly different from high-familiarity/high-frequency (HH) sentences (Bent et al., 2022).^2^ Therefore, only word recognition accuracy of the medical sentences was analyzed. Sentence type was contrast-coded such that HH items were considered the baseline, and high-familiarity/low-frequency (HL) and low-familiarity/low-frequency (LL) items were compared to the HH baseline. Additionally, contrast codes for sentence type were set to compare LL items to HL items. Self-rated knowledge of medical terminology and the scores on the 15iSSQ were investigated but neither improved model fit. Participants’ rating of background noise in their environment was also investigated but also showed no improvement to the model fit. These variables are not discussed further.

The final model included age group, listening condition, and sentence type, as well as pairwise interactions between their effects and their three-way interactions. Random intercepts for participant and test item were included as random effects. We conducted a separate analysis investigating the influence of the composite medical experience score. Effect sizes are interpreted from the beta estimates (see Baguley, 2009). A summary of the linear mixed-effects model can be found in Appendix A.

### Results

**Fig. 4:**
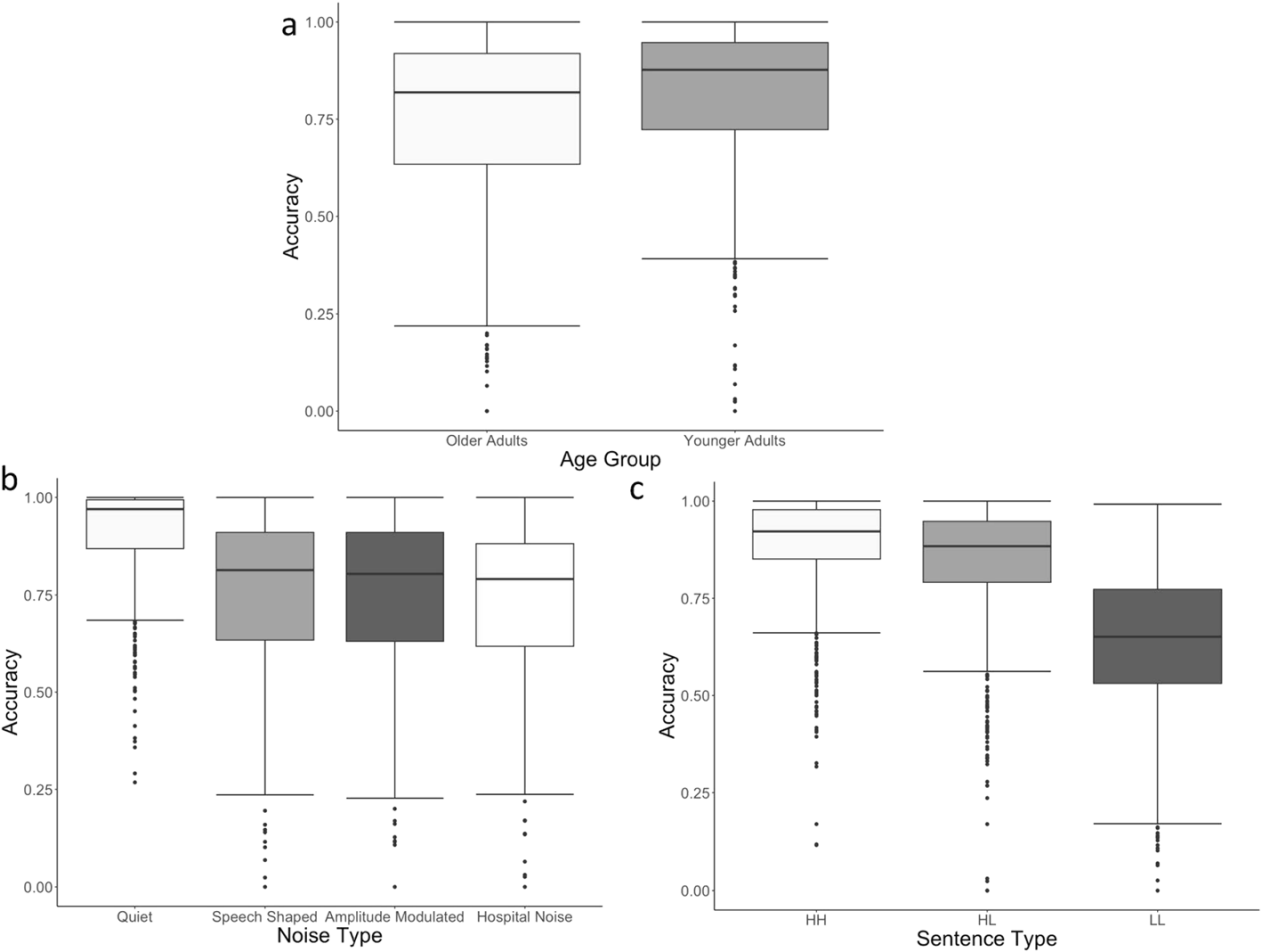
Panel **a** shows the effect of age comparing younger and older listeners, Panel **b** shows the effect of noise type comparing quiet, speech-shaped noise, amplitude-modulated hospital noise, and hospital noise, and Panel **c** shows the effect of sentence type: high familiarity/high frequency, high familiarity/low frequency, and low familiarity/low frequency

Figure 4 displays the main effects we explored first in the model.

Average TSR score (Accuracy) between the two age groups (younger and older adults) demonstrates that older adults had significantly lower word recognition accuracy overall compared to younger adults (*t* = 3.010, *p* = 0.003). Further, overall word recognition accuracy was significantly lower in any of the background noise conditions compared to the quiet condition (*t* = − 15.729, *p* < 0.001). Across both age groups, the accuracy in hospital noise is also lower than in the two speech-shaped noise conditions (*t* = 1.970, *p* = 0.049). While this result does not demonstrate conclusively that hospital noise is “worse” than the other types of noise that have been studied in more detail, it does suggest that it is at least as bad as these types of noise. Word recognition accuracy was not significantly different in the AM speech-shaped noise compared to the standard speech-shaped noise (*t* = 0.118, *p* = 0.906).

Across sentence types, accuracy on HH stimuli was significantly higher than on the HL and LL stimuli (*t* = − 15.687, *p* < 0.001), and performance on HL stimuli was significantly more accurate than on LL stimuli (*t* = − 19.152, *p* < 0.001). These results align with previous findings from Bent et al. (2022), which demonstrated that listeners could better identify more frequent words than less frequent words and more familiar words than less familiar words.

**Fig. 5:**
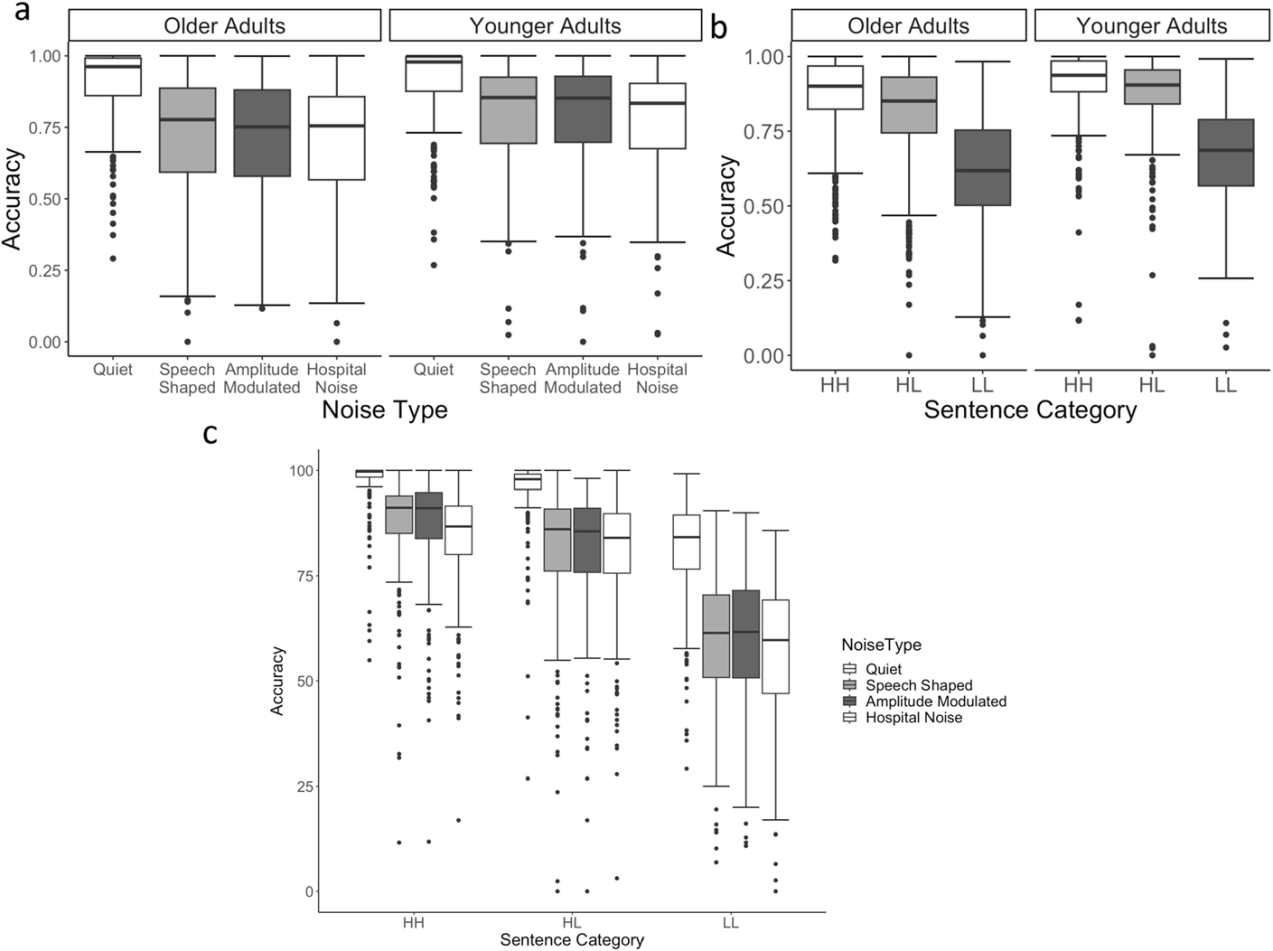
Panel **a** shows the interaction between age and noise type, Panel **b** shows the interaction between age and sentence type, and Panel **c** shows the interaction between age and noise type the interaction between noise type and sentence type

Figure 5 displays the significant two-way interactions in the model.

The interaction between type of background noise and sentence type was such that the comparison between quiet conditions and the three noise types differentially affected the low-frequency and low-familiarity sentences. That is, the interaction between the quiet and noise conditions for the high-familiarity and high-frequency sentences compared to the other types of sentences is significant (*t* = − 3.577, *p* < 0.001), as is the interaction between quiet and noise conditions for the high-familiarity/low-frequency sentences compared to the low-frequency/low-familiarity sentences (*t* = − 3.647, *p* < 0.001). None of the other contrast-coded comparisons between noise type and sentence type were significant (all *t*s < 1, all *p* > 0.35). These results suggest that the challenges of listening to sentences with lower frequency and lower familiarity words on word recognition accuracy was exacerbated in any presence of background noise compared to in quiet and compared to high-frequency stimuli and high-familiarity stimuli; however, this effect was not dependent on the specific characteristics of background noise.

Older adults’ accuracy was significantly more affected by any type of background noise, relative to the quiet condition, compared to younger adults. That is, the interaction between age and the noise was significant for the quiet condition compared to all the noise conditions (*t* = 9.916, *p* < 0.001). No other contrast-coded comparisons were significant (all *t*s < 1, all *p* > 0.6).

Older and younger adults showed no significant difference in word recognition accuracy decrements between HH stimuli and HL and LL stimuli (*t* = 1.591, *p* = 0.112). There was, however, a significant difference in performance decrements between HL stimuli and LL stimuli, with older adults showing a greater difference between the two sentence types than younger adults across all noise conditions (*t* = − 3.510, *p* < 0.001). This interaction indicates that older listeners were more affected by lower familiarity stimuli than younger listeners, but not necessarily by lower frequency items.

**Fig. 6:**
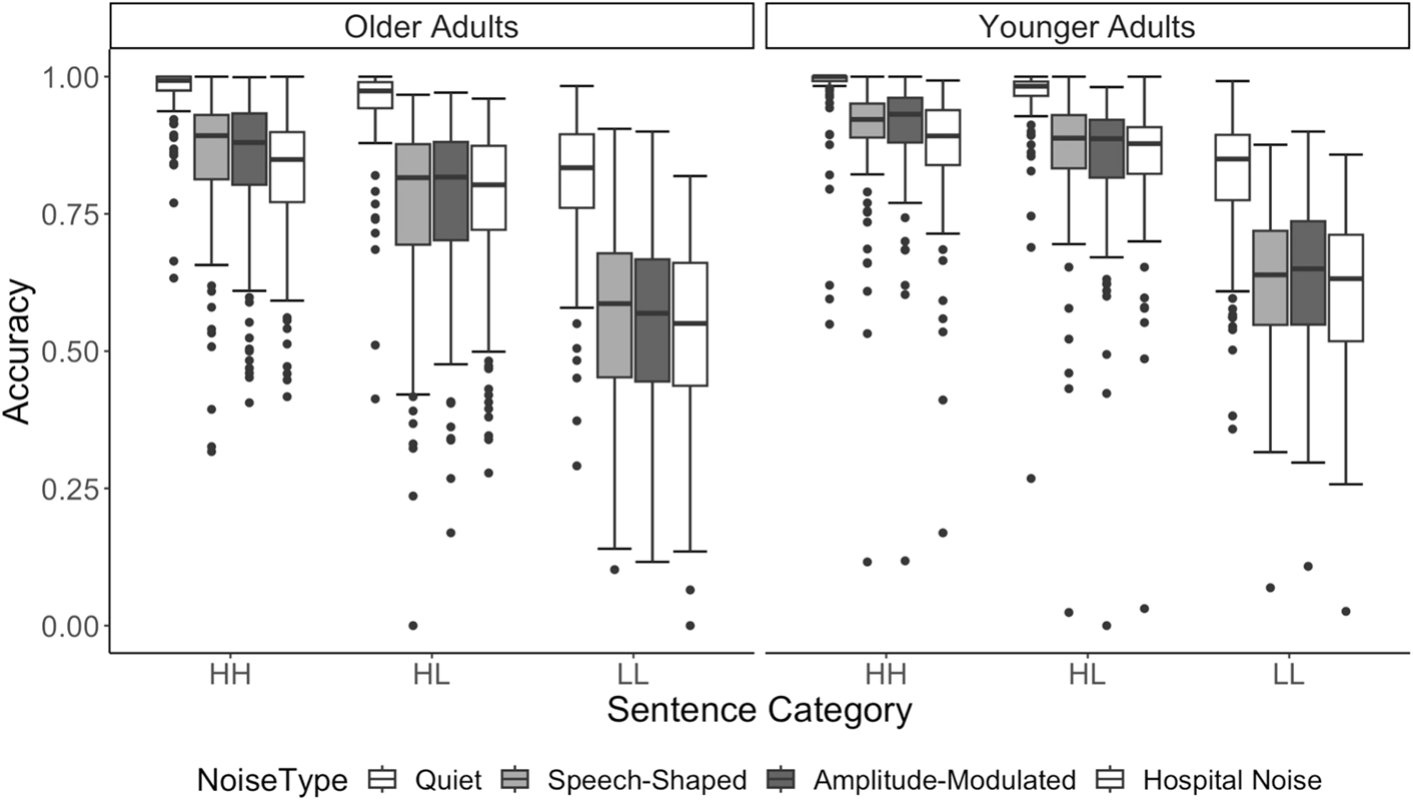
Accuracy by younger adults (left) and older adults (right) is shown for the four listening conditions (noise types) and sentence types on the x-axis

The three-way interaction among age group, sentence type, and type of background noise condition is illustrated in Fig. 6. There is a significant three-way interaction between sentence type, age group, and listening condition when investigating the quiet condition compared to the noise conditions and when comparing the HH sentences to the HL and LL sentences (*t* = 2.312, *p* = 0.021). In other words, although all participants showed significantly worse accuracy on lower frequency or lower familiarity sentences compared to baseline (HH) and this effect was greater in noise than in quiet, this interaction had a stronger impact on older adults’ accuracy compared to younger adults. Additionally, older adults’ show a significantly greater decrease in accuracy from HL items to LL items compared to younger adults in the presence of hospital noise compared to both speech-shaped noise conditions (*t* = − 2.041, *p* = 0.04). This result suggests that relative to younger adults, older adults found it particularly difficult to accurately understand sentences with low-familiarity and low-frequency medical words in the presence of hospital noise. The other three-way comparisons were not significant.

In summary, these results suggest that older adults had more difficulty correctly transcribing sentences that were heard in any type of background noise compared to younger adults. Older listeners had an especially difficult time on word recognition accuracy for sentences with low-frequency and low-familiarity words in noise. Although younger listeners also experienced difficulty, the decrements in accuracy from the HH stimuli were not as substantial for younger listeners as for the older group. Further, hospital noise seemed to more negatively impact older adults’ abilities to transcribe sentences with low-familiarity words than other types of noise, while younger listeners were equally affected by all types of noise on low-familiarity items and to a lesser extent than older listeners.

When investigating how recent medical experience relates to intelligibility performance, three findings emerged. Specifically, the interaction of noise type (hospital noise vs. other noise types) and medical experience is a significant predictor of model fit (*t* = 2.308, *p* = 0.021). Further, the three-way interaction between noise type (hospital vs. other noise types) and low-frequency and low-familiarity words is also a significant predictor of model fit (*t* = 2.507, *p* = 0.0122), suggesting that medical experience can support perception of speech in hospital noise, especially for low-frequency and low-familiarity words.

### General discussion

This study applied foundational concepts in hearing science and speech perception to investigate two potential challenges for successful communication in hospital settings between healthcare providers and patients: noise and less familiar/frequent terminology. Both younger and older adults had more difficulty understanding speech in a hospital noise condition compared to a speech-shaped noise condition or an amplitude-modulated speech-shaped noise and showed particularly poor performance when any type of noise was combined with low-familiarity, low-frequency words. These are the precise conditions in which important medical information may be conveyed and suggest that there is substantial opportunity for miscommunication between healthcare providers and patients.

Although both older and younger adults had more difficulty in the noise conditions than quiet conditions, consistent with decades of speech-in-noise literature, older adults had more difficulty in noise than the younger adults in Experiment 3. Furthermore, the hospital noise condition appeared to be particularly detrimental for perception. In Experiment 2, older adults had more difficulty with the hospital noise condition than the speech-shaped noise condition and in Experiment 3, considered together younger and older adults had more difficulty with the hospital noise condition compared to either of the speech-shaped noise conditions. The result deviates from the findings in Bent et al. (2022) which suggested that speech-shaped noise and hospital noise caused similar levels of challenge for young adults. We offer caution in over-interpreting the result that hospital noise is worse than other types of noise, as the analysis includes multiple interactions, and this result could be a “false-positive”. It is clear across the two experiments and our previous work that hospital noise is just as challenging as more well-studied types of noise. Further, the design of this study had noise as a within-participants variable compared to our previous study in which noise condition was an across-participants variable. Therefore, the result in our previous study of similar performance in hospital noise vs. speech-shaped noise may have been influenced by across participant group factors. Experiments 2 and 3 also varied in the types of variability listeners had to contend with. Listeners in Experiment 2 were handling variability across talkers, but noise type was constant, and those in Experiment 3 had the reverse, with variability in noise types, but a single talker. Although this work is a replication in some ways of older adults’ challenges with speech in noise, we have extended these findings to a new ecologically valid noise source.

Studies that compare the impacts of informational vs. energetic masking frequently use speech (single or multiple talkers) as the noise source that contributes to informational masking. Other types of soundscapes deserve attention as well, particularly because the sounds included in some of them may be highly attention grabbing (e.g., medical alarms in hospitals or sirens in traffic noise) but would not introduce competing linguistic information. The results here suggest that communication challenges present in hospitals are not just about energetic masking, but that the informational components are essential to consider for architectural engineers since it is not merely the presence of noise or the fluctuations in the noise source, but the information present in these signals that leads to challenges for speech communication.

We have begun here to disentangle how masker fluctuations and information in hospital soundscapes contribute to intelligibility. Specifically, the results from Experiment 3 suggest that masker fluctuations are not the primary contributor to differences across listening conditions, but rather identifiable information in hospital noise is the source of the listening challenge. Further work should be conducted with hospital noises that have different characteristics. It will be important to investigate hospital noise sources with and without voices (with identifiable semantic content or not) as well as hospital noise in which the environmental sounds are more or less identifiable. These ecologically valid noise sources would not only provide new knowledge about how different types and sources of informational masking impact speech understanding at different points in the lifespan but would also provide important information about how different noises in hospital soundscapes impact speech understanding. This knowledge would be useful for acoustic engineers and healthcare professionals alike.

Although the hospital noise condition was the most challenging for listeners, participants with more extensive experience with hospitals appeared to be better equipped to understand speech with these types of maskers, suggesting that long-term, real world experience with the noise source may benefit speech perception. Our composite measure of listeners’ experiences in healthcare settings improved model fit and showed that healthcare experience was particularly beneficial for hospital noise and for sentences with low-frequency/familiarity medical terminology. This finding suggests that listeners may be able to adapt to specific types of noise to facilitate word recognition. These listeners may also have greater knowledge of medical terminology gained through their real-world experience in hospital settings that supports their speech-in-noise performance with sentences containing terminology that is less familiar/frequency for most listeners.

The finding of better performance in hospital noise for those with more experience in these settings builds on prior research showing that listeners can adapt to talker-related signal differences after both short- and long-term exposure (e.g., second language accents; Bent & Baese-Berk, 2021) and to noise after short term exposure (Bent et al., 2009; Felty et al., 2009). However, there is little evidence regarding long-term adaptation to noise in terms of speech perception benefits. For example, in Pope et al. (2013), the number of hospitalizations, a measure of experience with hospital noise, did not impact speech understanding or recall in hospital noise. Other impacts (e.g., tendency to focus attention) of long-term exposure to noise also suggest little long-term adaptation (Brown & van Kamp, 2009; Weinstein, 1982). Our findings suggest that there may indeed be long-term adaptation to specific noise sources. Listener experience with hospital noise may not only arise only from hospital stays but also from other hospital experiences (e.g., visiting extensively with a loved one who is hospitalized or working in a hospital). Our composite score for healthcare experience included not just the listeners’ hospital stays but more broadly their time spent in healthcare facilities as patients, caregivers, or visitors. Considering the relative dearth of work in this area, this question deserves additional attention. The perceived challenges of communicating in hospital noises for patients may be underestimated by healthcare professionals because they have extensive experience in these soundscapes and have been able to adapt.

In addition to the noise characteristics, word recognition performance was impacted by both word familiarity and frequency. In quiet, older adults had high word recognition accuracy for sentences with high-familiarity words that varied in frequency, but showed substantial accuracy decreases for sentences with low-familiarity words. The impact of word frequency for high-familiarity words supports prior findings showing similar effects of lexical variables, such as word frequency, throughout the lifespan (Taler et al., 2010). Although the findings for word frequency replicate prior literature, it is worth noting that it is extremely uncommon to include words in intelligibility tests that are lower in familiarity. For example, speech-in-noise testing in both research and clinical settings typically uses words that are high in familiarity although they may vary substantially in frequency (Bell & Wilson, 2001; Gilbert et al., 2013; Nilsson et al., 1994; Schafer et al., 2012). Although a few of the words included in this set received very low-familiarity ratings from the older listener cohort in the word familiarity rating task (e.g., ototoxic = 1.88; kyphosis = 1.41; peccant = 1.61), the average rating from older adults for words in the low-familiarity, low-frequency condition was 5.12 (Standard Dev = 1.64). A rating of 5 corresponds to the descriptions of “*You are certain that you have seen the word, but you only have a vague idea of its meaning.”* on the familiarity rating scale. Words of this type are certainly encountered in medical settings during diagnoses and treatment plans. Further, patients are highly likely to encounter words that they would rate as a “1” (“You have never seen or heard this word before”), when, for instance, prescribed a medication with a completely unfamiliar name. Understanding how these words are perceived in different acoustic environments is important for medical contexts as well as other contexts in which listeners are presented with new words (e.g., educational settings). Furthermore, there is evidence that healthcare providers do not consistently use “everyday language” with patients (Bourhis et al., 1989; Denton et al., 2020) and that they overestimate how much of the information they provide is understood by patients (Byrne & Edeani, 1984; Yoshida & Yoshida, 2014). Even words that are high in familiarity but lower in frequency are more difficult to recall (Balota & Neely, 1980), which could have implications for accurate adherence of discharge instructions or full understanding of a diagnosis.

Communication in hospitals provides an important application of theoretical models of speech communication, such as the Ease of Language Understanding (ELU) model (Rönnberg et al., 2013) or the Framework for Understanding Effortful Listening (FUEL) (Pichora-Fuller et al., 2016). The ELU, for example, suggests that communication in hospitals is likely to require greater recruitment of working memory capacity, due to the environmental noise that will likely inhibit some speech from being processed rapidly and implicitly but will rather draw on explicit processing mechanisms. The decreases in working memory capacity found in older adults may thus further limit their ability to understand health information presented to them. In addition to the application of these concepts in the model, our results suggest that more consideration should be made regarding the strength of encoding of the semantic information in long-term memory. That is, if explicit processing of auditory information is required due to adverse listening conditions, listeners will be required to explicitly draw upon semantic long-term memory. However, less familiar or frequently encountered words may have weaker phonological-lexical representations further taxing the working memory system required to access the correct lexical entry. Even if the content of the message is accurately apprehended, these input related factors will require substantially greater cognitive resources and listening effort.

Older adults are not the only listeners who have greater challenges understanding speech in noisy environments. Listeners from other groups who have well-known challenges with speech in noise—such as bilinguals (Rogers et al., 2006), those interacting in a second language (e.g., Bent & Bradlow, 2003), and children (Fallon et al., 2000)—should also be tested in medical soundscapes. Likewise, perception of speech produced by talkers with other characteristics, including those who have less familiar accents (both regional first language accents and second language accents) should also be assessed within medical noise conditions with sentences that have words that vary in familiarity and frequency. The combinations of multiple factors related to both the characteristics of the listener and the talker could lead to even greater decrements for understanding healthcare providers’ speech.

In addition to investigating other populations of listeners and talkers, future work should focus on methods for ameliorating the effects of noise and unfamiliar terminology on possible miscommunications in healthcare settings. The methods used to improve communication could come from several fronts. First, it is now well-established that hospitals are noisy and acoustically challenging places. Thus, a focus on improving the soundscapes is warranted. Alarm noise has been particularly problematic historically and can result in alarm fatigue for care providers (Albanowski et al., 2023; Oleksy & Schlesinger, 2019); therefore, improving alarm environments could be one critical approach. Other strategies may include administrative protocols (e.g., quiet time, education) and engineered solutions (e.g., sound absorption, adjusted layout, reduce noise sources) (Busch-Vishniac & Ryherd, 2023). Second, medical providers could receive additional training and continuing education on the use of everyday language when communicating with patients. If there are specific medical terms that must be used (e.g., medication names, diagnostic terminology), there should be clear, thorough explanations provided for those terms including ensuring definition or explanation of terms are provided in writing following appointment or discharge. Third, healthcare providers should be aware that patients may have more difficulty understanding them in noisy environments if they have never met the patient before, but that adaptation to unfamiliar talkers and accents can happen relatively quickly (Bradlow & Bent, 2008). In addition to building rapport, some initial time speaking with the patient about less critical health information could allow the patient to adapt to the healthcare provider’s speech production patterns before critical information is presented.

### Limitations

There are several limitations to this study. First, because we completed the data collection online, it was not possible to assess participants’ hearing beyond the speech spatial and qualities of hearing scale (15iSSQ) questionnaire and inclusion of the headphone screening. The 15iSSQ has been shown to relate to hearing loss as measured by pure-tone thresholds (Moulin et al., 2019) and therefore was included as a measure of how hearing loss may contribute to performance in the intelligibility task. However, the inclusion of 15iSSQ scores did not improve model fit. In line with that result, only a few of our participants indicated that they had hearing loss, but without audiometric testing, we could not precisely measure hearing thresholds. Future studies should incorporate full audiograms to better characterize the participants’ hearing abilities and investigate how hearing thresholds influence performance across noise conditions; these measures would allow us to fully disentangle other age-related changes from the potential presence of hearing loss. Measures of other cognitive and linguistic abilities known to influence performance in speech-in-noise tests (Füllgrabe et al., 2015; Humes et al., 2013; Moore et al., 2014) should also be incorporated in future studies. Explicit tests of medical terminology knowledge could be incorporated into the protocol, which may help to explain variance among the listeners.

Finally, this study only investigated word identification without measures of comprehension or memory. Although recognizing words is an essential first step for successful communication without which higher level processing or retention of messages cannot take place, future work should incorporate comprehension measures, such as extracting main ideas from conversational exchanges with healthcare providers. Furthermore, memory measures should be investigated as it has been shown that perception of speech in noisy conditions impacts retention of information, even when the speech was accurately perceived (Rabbitt, 1968). Studies investigating these issues will be important for understanding how noisy environments may interfere with adherence to medical discharge instructions. Even if information presented in noisy hospital settings is understood initially, patients may have difficulty recalling instruction details at later points in time (e.g., remembering details about medication regimens when they are home from the hospital). The understanding and retention of important details of these interactions will provide a richer understanding of how the acoustic environment may impact communication success between providers and patients.

## Conclusion

In surveys of patient–provider communication, older patients frequently report communication difficulties, including challenges resulting from background noise (A. Shukla et al., 2019). The signal-to-noise ratio used in this study approximates expected signal-to-noise ratios in hospitals when speech is produced at a typical conversational level (Pope et al., 2013). These results provide a starting point for understanding how the hospital soundscape may impact healthcare provider–patient communication for older adults, which ultimately can influence health outcomes, particularly when interacting in noisy, high-stress environments like hospitals. This study highlights the challenges that older adults may face during interactions with healthcare providers, when adverse conditions are combined, as with noisy hospital settings and less familiar medical terminology.

## Appendix

Summary of the linear mixed-effects model results in Experiment 3. HH refers to sentences with high-familiarity and high-frequency words; HL for sentences with high-familiarity and low-frequency words; LL for sentences with low-familiarity and low-frequency words.

**Table:**

Predictors	**Accuracy**		
	Estimates	CI	*p*
(Intercept)	0.81	0.79–0.82	**< 0.001**
Older adults versus younger adults (OA vs. YA)	0.02	0.01–0.03	**0.003**
Quiet versus noise	− 0.04	− 0.04–− 0.03	**< 0.001**
Hospital noise versus both speech-shaped noises	0.01	0.00–0.01	**0.049**
Standard speech-shaped versus AM speech-shaped	0.00	− 0.01–0.01	0.906
HH versus other	− 0.05	− 0.05–− 0.04	**< 0.001**
HL versus LL	− 0.10	− 0.11–− 0.09	**< 0.001**
OA versus YA × quiet versus noise	0.01	0.00–0.01	**< 0.001**
OA versus YA × hospital versus both speech-shaped	0.00	− 0.00–0.00	0.765
OA versus YA × speech-shaped versus AM speech-shaped	− 0.00	− 0.00–0.00	0.992
OA versus YA × HH versus other	0.00	− 0.00–0.00	0.112
OA versus YA × HL versus LL	− 0.00	− 0.01–− 0.00	**< 0.001**
Quiet versus noise × HH versus other	− 0.01	− 0.01–− 0.00	**< 0.001**
Hospital versus both speech-shaped × HH versus other	− 0.00	− 0.01–0.00	0.364
Speech-shaped versus AM speech-shaped × HH versus other	0.00	− 0.01–0.01	0.723
Quiet versus noise × HL versus LL	− 0.01	− 0.02–− 0.01	**< 0.001**
Hospital versus both speech-shaped × HL versus LL	0.00	− 0.01–0.01	0.682
Speech-shaped versus AM speech-shaped × HL versus LL	0.00	− 0.01–0.02	0.828
OA versus YA × quiet versus noise × HH versus other	0.00	0.00–0.00	**0.021**
OA versus YA × hospital versus both speech-shaped × HH versus other	− 0.00	− 0.00–0.00	0.362
OA versus YA × speech-shaped versus AM speech-shaped × HH versus Other	0.00	− 0.00–0.00	0.385
OA versus YA × quiet versus noise × HL versus LL	0.00	− 0.00–0.00	0.493
OA versus YA × hospital versus both speech-shaped × HL versus LL	− 0.00	− 0.00–− 0.00	**0.041**
OA versus YA × speech-shaped versus AM speech-shaped × HL versus LL	− 0.00	− 0.01–0.00	0.137

Significant effects are shown in bold

## Footnotes

1 Pearson’s correlation coefficients can be interpreted as effect sizes, and this coefficient is indicative of a large effect (Cohen, 1988).

2 An analysis of our current data demonstrates that there is not a significant difference between the standardized speech sentences and HH sentences; therefore, we proceed with the analysis of just the medical sentences here.

## References

[CR1] Adel Ghahraman, M., Ashrafi, M., Mohammadkhani, G., & Jalaie, S. (2020). Effects of aging on spatial hearing. *Aging Clinical and Experimental Research,**32*(4), 733–739. 10.1007/s40520-019-01233-3 31203530 10.1007/s40520-019-01233-3

[CR2] Albanowski, K., Burdick, K. J., Bonafide, C. P., Kleinpell, R., & Schlesinger, J. J. (2023). Ten years later, alarm fatigue is still a safety concern. *AACN Advanced Critical Care,**34*(3), 189–197. 10.4037/aacnacc2023662 37644627 10.4037/aacnacc2023662

[CR3] Anderson, S., Parbery-Clark, A., Yi, H. G., & Kraus, N. (2011). A neural basis of speech-in-noise perception in older adults. *Ear and Hearing,**32*(6), 750–757. 10.1097/AUD.0b013e31822229d3 21730859 10.1097/AUD.0b013e31822229d3 PMC3189261

[CR4] Baguley, T. (2009). Standardized or simple effect size: What should be reported? *British Journal of Psychology,**100*, 603–617. 10.1348/000712608X377117 19017432 10.1348/000712608X377117

[CR5] Balota, D. A., & Neely, J. H. (1980). Test-expectancy and word-frequency effects in recall and recognition. *Journal of Experimental Psychology: Human Learning and Memory,**6*(5), 576–587. 10.1037/0278-7393.6.5.576

[CR6] Balota, D. A., Pilotti, M., & Cortese, M. J. (2001). Subjective frequency estimates for 2,938 monosyllabic words. *Memory and Cognition,**29*(4), 639–647. 10.3758/bf03200465 11504012 10.3758/bf03200465

[CR7] Bell, R., & Buchner, A. (2007). Equivalent irrelevant-sound effects for old and young adults. *Memory and Cognition,**35*(2), 352–364. 10.3758/BF03193456 17645176 10.3758/bf03193456

[CR8] Bell, T. S., & Wilson, R. H. (2001). Sentence recognition materials based on frequency of word use and lexical confusability. *Journal of the American Academy of Audiology,**12*(10), 514. 11791938

[CR9] Ben-David, B. M., Tse, V. Y. Y., & Schneider, B. A. (2012). Does it take older adults longer than younger adults to perceptually segregate a speech target from a background masker? *Hearing Research,**290*(1), 55–63. 10.1016/j.heares.2012.04.022 22609772 10.1016/j.heares.2012.04.022

[CR10] Bent, T., & Baese-Berk, M. (2021). Perceptual learning of accented speech. *The Handbook of Speech Perception* (pp. 428–464). John Wiley & Sons, Ltd. 10.1002/9781119184096.ch16

[CR11] Bent, T., Baese-Berk, M. M., Ryherd, E. E., & Perry, S. (2022). Intelligibility of medically related sentences in quiet, speech-shaped noise, and hospital noise. *Journal of the Acoustical Society of America,**151*(5), 3496–3508. 10.1121/10.0011394 35649935 10.1121/10.0011394

[CR12] Bent, T., & Bradlow, A. R. (2003). The interlanguage speech intelligibility benefit. *The Journal of the Acoustical Society of America,**114*(3), 1600–1610. 10.1121/1.1603234 14514213 10.1121/1.1603234

[CR13] Bent, T., Buchwald, A., & Pisoni, D. B. (2009). Perceptual adaptation and intelligibility of multiple talkers for two types of degraded speech. *Journal of the Acoustical Society of America,**126*(5), 2660–2669. 10.1121/1.3212930 19894843 10.1121/1.3212930 PMC2787077

[CR14] Blustein, J., Weinstein, B. E., & Michael, J. C. (2018). Tackling hearing loss to improve the care of older adults. *BMJ: British Medical Journal,**360*, 1–4. 10.1136/bmj.k21 29348197

[CR15] Bosker, H. R. (2021). Using fuzzy string matching for automated assessment of listener transcripts in speech intelligibility studies. *Behavior Research Methods,**53*(5), 1945–1953. 10.3758/s13428-021-01542-4 33694079 10.3758/s13428-021-01542-4 PMC8516752

[CR16] Bourhis, R. Y., Roth, S., & MacQueen, G. (1989). Communication in the hospital setting: A survey of medical and everyday language use amongst patients, nurses and doctors. *Social Science and Medicine,**28*(4), 339–346. 10.1016/0277-9536(89)90035-x 2705006 10.1016/0277-9536(89)90035-x

[CR17] Bradlow, A. R., & Bent, T. (2008). Perceptual adaptation to non-native speech. *Cognition,**106*(2), 707–729. 10.1016/j.cognition.2007.04.005 17532315 10.1016/j.cognition.2007.04.005 PMC2213510

[CR18] Brown, V. A., Van Engen, K. J., & Peelle, J. E. (2021). Face mask type affects audiovisual speech intelligibility and subjective listening effort in young and older adults. *Cognitive Research: Principles and Implications,**6*(1), 1–12. 10.1186/s41235-021-00314-0 34275022 10.1186/s41235-021-00314-0 PMC8286438

[CR19] Brown, A. L., & van Kamp, I. (2009). Response to a change in transport noise exposure: A review of evidence of a change effect. *The Journal of the Acoustical Society of America,**125*(5), 3018–3029. 10.1121/1.3095802 19425645 10.1121/1.3095802

[CR20] Brungart, D. S. (2001). Informational and energetic masking effects in the perception of two simultaneous talkers. *The Journal of the Acoustical Society of America,**109*(3), 1101–1109. 10.1121/1.1345696 11303924 10.1121/1.1345696

[CR21] Brysbaert, M., & New, B. (2009). Moving beyond Kucera and Francis: A critical evaluation of current word frequency norms and the introduction of a new and improved word frequency measure for American English. *Behavioral Research Methods,**41*(4), 977–990. 10.3758/BRM.41.4.977 10.3758/BRM.41.4.977 19897807

[CR22] Busch-Vishniac, I. (2019). *Next steps in hospital noise research*. Universitätsbibliothek der RWTH Aachen

[CR23] Busch-Vishniac, I., & Ryherd, E. (2023). Hospital soundscapes. In B. Schulte-Fortkamp, A. Fiebig, J. Sisneros, A. Popper, & R. Fay (Eds.), *Springer Handbook of Auditory Research. * (Vol. 76). Springer Publishing.

[CR24] Busch-Vishniac, I., West, J. E., Barnhill, C., Hunter, T., Orellana, D., & Chivukula, R. (2005). Noise levels in Johns Hopkins Hospital. *Journal of the Acoustical Society of America,**118*(6), 3629–3645. 10.1121/1.2118327 16419808 10.1121/1.2118327

[CR25] Byrne, T. J., & Edeani, D. (1984). Knowledge of medical terminology among hospital patients. *Nursing Research,**33*(3), 178–181. 6563536

[CR26] Carroll, R., Warzybok, A., Kollmeier, B., & Ruigendijk, E. (2016). Age-related differences in lexical access relate to speech recognition in noise. *Frontiers in Psychology,**7*, 990. 10.3389/fpsyg.2016.00990 27458400 10.3389/fpsyg.2016.00990 PMC4930932

[CR27] Chang, J. E., Weinstein, B., Chodosh, J., & Blustein, J. (2018). Hospital readmission risk for patients with self-reported hearing loss and communication trouble. *Journal of the American Geriatrics Society,**66*(11), 2227–2228. 10.1111/jgs.15545 30289969 10.1111/jgs.15545

[CR28] Christ, S. E., White, D. A., Mandernach, T., & Keys, B. A. (2001). Inhibitory control across the life span. *Developmental Neuropsychology,**20*(3), 653–669. 10.1207/S15326942DN2003_7 12002099 10.1207/S15326942DN2003_7

[CR29] Connine, C. M., Mullennix, J., Shernoff, E., & Yelen, J. (1990). Word familiarity and frequency in visual and auditory word recognition. *Journal of Experimental Psychology: Learning, Memory, and Cognition,**16*(6), 1084–1096. 10.1037/0278-7393.16.6.1084 2148581 10.1037//0278-7393.16.6.1084

[CR30] Cooke, M. (2006). A glimpsing model of speech perception in noise. *Journal of the Acoustical Society of America,**119*(3), 1562–1573. 10.1121/1.2166600 16583901 10.1121/1.2166600

[CR31] Cruickshanks, K. J., Zhan, W., & Zhong, W. (2010). Epidemiology of age-related hearing impairment. In S. Gordon-Salant, R. D. Frisina, A. N. Popper, & R. R. Fay (Eds.), *The Aging Auditory System* (pp. 259–274). Springer. 10.1007/978-1-4419-0993-0_9

[CR32] Darbyshire, J. L., & Young, J. D. (2013). An investigation of sound levels on intensive care units with reference to the WHO guidelines. *Critical Care,**17*(5), R187. 10.1186/cc12870 24005004 10.1186/cc12870 PMC4056361

[CR33] Davis, A., McMahon, C. M., Pichora-Fuller, K. M., Russ, S., Lin, F., Olusanya, B. O., Chadha, S., & Tremblay, K. L. (2016). Aging and hearing health: The life-course approach. *The Gerontologist,**56*(Suppl_2), S256–S267. 10.1093/geront/gnw033 26994265 10.1093/geront/gnw033 PMC6283365

[CR34] De Lima Andrade, E., Da Cunha, E., Silva, D. C., De Lima, E. A., De Oliveira, R. A., Zannin, P. H. T., & Martins, A. C. G. (2021). Environmental noise in hospitals: A systematic review. *Environmental Science and Pollution Research,**28*(16), 19629–19642. 10.1007/s11356-021-13211-2 33674976 10.1007/s11356-021-13211-2 PMC7935697

[CR35] Denton, C. P., Laird, B., Moros, L., & Flores, J. L. L. (2020). Challenges in physician-patient communication for optimal management of systemic sclerosis-associated interstitial lung disease: A discourse analysis. *Clinical Rheumatology,**39*(10), 2989–2998. 10.1007/s10067-020-05063-x 32285258 10.1007/s10067-020-05063-x PMC7497349

[CR36] Dorot, D., & Mathey, S. (2010). Visual word recognition in young and older adults: A study of cohort effects for lexical variables. *European Review of Applied Psychology,**60*(3), 163–172. 10.1016/j.erap.2010.02.001

[CR37] Dubno, J. R., Dirks, D. D., & Morgan, D. E. (1984). Effects of age and mild hearing-loss on speech recognition in noise. *Journal of the Acoustical Society of America,**76*(1), 87–96. 10.1121/1.391011 6747116 10.1121/1.391011

[CR38] Dubno, J. R., Horwitz, A. R., & Ahlstrom, J. B. (2002). Benefit of modulated maskers for speech recognition by younger and older adults with normal hearing. *Journal of the Acoustical Society of America,**111*(6), 2897–2907. 10.1121/1.1480421 12083223 10.1121/1.1480421

[CR39] Dubno, J. R., Horwitz, A. R., & Ahlstrom, J. B. (2003). Recovery from prior stimulation: Masking of speech by interrupted noise for younger and older adults with normal hearing. *Journal of the Acoustical Society of America,**113*, 2084–2094. 10.1121/1.1555611 12703719 10.1121/1.1555611

[CR40] Fallon, M., Trehub, S. E., & Schneider, B. A. (2000). Children’s perception of speech in multitalker babble. *The Journal of the Acoustical Society of America,**108*(6), 3023–3029. 10.1121/1.1323233 11144594 10.1121/1.1323233

[CR41] Felty, R. A., Buchwald, A., & Pisoni, D. B. (2009). Adaptation to frozen babble in spoken word recognition. *The Journal of the Acoustical Society of America,**125*(3), EL93–EL97. 10.1121/1.3073733 19275281 10.1121/1.3073733 PMC4109289

[CR42] Festen, J. M., & Plomp, R. (1990). Effects of fluctuating noise and interfering speech on the speech-reception threshold for impaired and normal hearing. *Journal of the Acoustical Society of America,**88*(4), 1725–1736. 10.1121/1.400247 2262629 10.1121/1.400247

[CR43] Fortenbaugh, F. C., DeGutis, J., Germine, L., Wilmer, J. B., Grosso, M., Russo, K., & Esterman, M. (2015). Sustained attention across the life span in a sample of 10,000: Dissociating ability and strategy. *Psychological Science,**26*(9), 1497–1510. 10.1177/0956797615594896 26253551 10.1177/0956797615594896 PMC4567490

[CR44] Füllgrabe, C., Moore, B. C. J., & Stone, M. A. (2015). Age-group differences in speech identification despite matched audiometrically normal hearing: Contributions from auditory temporal processing and cognition. *Frontiers in Aging Neuroscience,**6*, 347. 10.3389/fnagi.2014.00347 25628563 10.3389/fnagi.2014.00347 PMC4292733

[CR45] Gatehouse, S., & Noble, W. (2004). The speech, spatial and qualities of hearing scale (SSQ). *International Journal of Audiology,**43*(2), 85–99. 10.1080/14992020400050014 15035561 10.1080/14992020400050014 PMC5593096

[CR46] George, J., Bleasdale, S., & Singleton, S. J. (1997). Causes and prognosis of delirium in elderly patients admitted to a district general hospital. *Age and Ageing,**26*(6), 423–427. 10.1093/ageing/26.6.423 9466291 10.1093/ageing/26.6.423

[CR47] Gifford, R. H., Bacon, S. P., & Williams, E. J. (2007). An examination of speech recognition in a modulated background and of forward masking in younger and older listeners. *Journal of Speech, Language and Hearing Research,**50*(4), 857–864. 10.1044/1092-4388(2007/060) 10.1044/1092-4388(2007/060) PMC2441836 17675591

[CR48] Gilbert, J. L., Tamati, T. N., & Pisoni, D. B. (2013). Development, reliability, and validity of PRESTO: A new high-variability sentence recognition test. *Journal of the American Academy of Audiology,**24*(1), 26–36. 10.3766/jaaa.24.1.4 23231814 10.3766/jaaa.24.1.4 PMC3683852

[CR49] Gladd, D. K., & Saunders, G. H. (2011). Ambient noise levels in the chemotherapy clinic. *Noise & Health,**13*(55), 444–451. 10.4103/1463-1741.90322 22122961 10.4103/1463-1741.90322 PMC4710469

[CR50] Gold, D. P., Andres, D., Etezadi, J., Arbuckle, T., Schwartzman, A., & Chaikelson, J. (1995). Structural equation model of intellectual change and continuity and predictors of intelligence in older men. *Psychology and Aging,**10*(2), 294–303. 10.1037/0882-7974.10.2.294 7662188 10.1037//0882-7974.10.2.294

[CR51] Harel-Arbeli, T., Wingfield, A., Palgi, Y., & Ben-David, B. M. (2021). Age-related differences in the online processing of spoken semantic context and the effect of semantic competition: Evidence from eye gaze. *Journal of Speech, Language, and Hearing Research,**64*(2), 315–327. 10.1044/2020_JSLHR-20-00142 33561353 10.1044/2020_JSLHR-20-00142

[CR52] Hartshorne, J. K., & Germine, L. T. (2015). When does cognitive functioning peak? The asynchronous rise and fall of different cognitive abilities across the life span. *Psychological Science,**26*(4), 433–443. 10.1177/0956797614567339 25770099 10.1177/0956797614567339 PMC4441622

[CR53] Helfer, K. S., & Freyman, R. L. (2008). Aging and speech-on-speech masking. *Ear and Hearing,**29*(1), 87–98. 10.1097/AUD.0b013e31815d638b 18091104 10.1097/AUD.0b013e31815d638b PMC2987598

[CR54] Helfer, K. S., Merchant, G. R., & Wasiuk, P. A. (2017). Age-related changes in objective and subjective speech perception in complex listening environments. *Journal of Speech, Language, and Hearing Research,**60*(10), 3009–3018. 10.1044/2017_JSLHR-H-17-0030 29049601 10.1044/2017_JSLHR-H-17-0030 PMC5945070

[CR55] Humes, L. (1996). Speech understanding in the elderly. *Journal of the American Academy of Audiology,**7*(3), 161–167. 8780988

[CR56] Humes, L., Kidd, G., & Lentz, J. (2013). Auditory and cognitive factors underlying individual differences in aided speech-understanding among older adults. *Frontiers in Systems Neuroscience,**7*, 55. 10.3389/fnsys.2013.00055 24098273 10.3389/fnsys.2013.00055 PMC3787592

[CR57] Kaandorp, M. W., De Groot, A. M. B., Festen, J. M., Smits, C., & Goverts, S. T. (2016). The influence of lexical-access ability and vocabulary knowledge on measures of speech recognition in noise. *International Journal of Audiology,**55*(3), 157–167. 10.3109/14992027.2015.1104735 26609557 10.3109/14992027.2015.1104735

[CR58] Kavé, G., & Halamish, V. (2015). Doubly blessed: Older adults know more vocabulary and know better what they know. *Psychology and Aging,**30*(1), 68–73. 10.1037/a0038669 25602490 10.1037/a0038669

[CR59] Kidd, G., Jr., Mason, C. R., Richards, V. M., Gallun, F. J., & Durlach, N. I. (2008). Informational masking. In W. Yost (Ed.), *Springer Handbook of Auditory Research, 29: Auditory Perception of Sound Sources* (pp. 143–190). New York: Springer.

[CR60] Klatte, M., Lachmann, T., Schlittmeier, S., & Hellbrück, J. (2010). The irrelevant sound effect in short-term memory: Is there developmental change? *European Journal of Cognitive Psychology,**22*(8), 1168–1191. 10.1080/09541440903378250

[CR61] Lin, F. R., Niparko, J. K., & Ferrucci, L. (2011). Hearing loss prevalence in the United States. *Archives of Internal Medicine,**171*(20), 1851–1852. 10.1001/archinternmed.2011.506 22083573 10.1001/archinternmed.2011.506 PMC3564588

[CR62] Lustig, C., Hasher, L., & Tonev, S. T. (2001). Inhibitory control over the present and the past. *European Journal of Cognitive Psychology,**13*(1–2), 107–122. 10.1080/09541440126215

[CR63] McAuliffe, M. J., Gibson, E. M. R., Kerr, S. E., Anderson, T., & LaShell, P. J. (2013). Vocabulary influences older and younger listeners’ processing of dysarthric speech. *Journal of the Acoustical Society of America,**134*(2), 1358–1368. 10.1121/1.4812764 23927132 10.1121/1.4812764

[CR64] McGinnis, D., & Zelinski, E. M. (2000). Understanding unfamiliar words: The influence of processing resources, vocabulary knowledge, and age. *Psychology and Aging,**15*(2), 335–350. 10.1037/0882-7974.15.2.335 10879587 10.1037//0882-7974.15.2.335

[CR65] McGinnis, D., & Zelinski, E. M. (2003). Understanding unfamiliar words in young, young-old, and old-old adults: Inferential processing and the abstraction-deficit hypothesis. *Psychology and Aging,**18*(3), 497–509. 10.1037/0882-7974.18.3.497 14518811 10.1037/0882-7974.18.3.497

[CR66] Messingher, G. (2013). “Relating hospital noise to staff outcomes in real and simulated settings,” (master’s thesis). Georgia Institute of Technology.

[CR67] Miller, G. A., & Licklider, J. C. R. (1950). The intelligibility of interrupted speech. *Journal of the Acoustical Society of America,**22*(2), 167–173. 10.1121/1.1906584

[CR68] Moore, D. R., Edmondson-Jones, M., Dawes, P., Fortnum, H., McCormack, A., Pierzycki, R. H., & Munro, K. J. (2014). Relation between speech-in-noise threshold, hearing loss and cognition from 40–69 years of age. *PLoS ONE,**9*(9), e107720. 10.1371/journal.pone.0107720 25229622 10.1371/journal.pone.0107720 PMC4168235

[CR69] Moulin, A., Vergne, J., Gallego, S., & Micheyl, C. (2019). A new speech, spatial, and qualities of hearing scale short-form: factor, cluster, and comparative analyses. *Ear and Hearing,**40*(4), 938–950. 10.1097/AUD.0000000000000675 30461444 10.1097/AUD.0000000000000675

[CR70] Nilsson, M., Soli, S. D., & Sullivan, J. A. (1994). Development of the Hearing In Noise Test for the measurement of speech reception thresholds in quiet and in noise. *Journal of the Acoustical Society of America,**95*(2), 1085–1099. 8132902 10.1121/1.408469

[CR71] Nusbaum, H. C., Pisoni, D. B., & Davis, C. K. (1984). *Sizing up the Hoosier Mental Lexicon: Measuring the Familiarity of 20,000 Words* (Research on Speech Perception Progress Report No. 10, pp. 357–376). Speech Research Laboratory Department of Psychology Indiana University.

[CR72] Oleksy, A. J., & Schlesinger, J. J. (2019). What’s all that noise-improving the hospital soundscape. *Journal of Clinical Monitoring and Computing,**33*(4), 557–562. 10.1007/s10877-018-0215-3 30390171 10.1007/s10877-018-0215-3

[CR73] Peirce, J., Gray, J. R., Simpson, S., MacAskill, M., Hochenberger, R., Sogo, H., Kastman, E., & Lindelov, J. K. (2019). PsychoPy2: Experiments in behavior made easy. *Behavior Research Methods,**51*(1), 195–203. 10.3758/s13428-018-01193-y 30734206 10.3758/s13428-018-01193-y PMC6420413

[CR74] Perry, S., Bent, T., Ryherd, E., & Baese-Berk, M. (2021) A novel corpus developed to evaluate the impact of hospital noise on speech intelligibility INTER-NOISE and NOISE-CON Congress and Conference Proceedings *263*(4), 2157–2163. 10.3397/IN-2021-2064

[CR75] Pichora-Fuller, M. K., Kramer, S. E., Eckert, M. A., Edwards, B., Hornsby, B. W., Humes, L. E., & Wingfield, A. (2016). Hearing impairment and cognitive energy: The framework for understanding effortful listening (FUEL). *Ear and Hearing,**37*, 5S-27S. 10.1097/AUD.0000000000000312 27355771 10.1097/AUD.0000000000000312

[CR76] Plomp, R. (1986). A signal-to-noise ratio model for the speech-reception threshold of the hearing impaired. *Journal of Speech, Language, and Hearing Research,**29*(2), 146–154. 10.1044/jshr.2902.146 10.1044/jshr.2902.146 3724108

[CR77] Pollack, I. (1975). Auditory informational masking. *Journal of the Acoustical Society of America,**57*, S5. 10.1121/1.1995329

[CR78] Pope, D. S., Gallun, F. J., & Kampel, S. (2013). Effect of hospital noise on patients’ ability to hear, understand, and recall speech. *Research in Nursing and Health,**36*(3), 228–241. 10.1002/nur.21540 23606205 10.1002/nur.21540

[CR79] Rabbitt, P. M. (1968). Channel-capacity, intelligibility and immediate memory. *Quarterly Journal of Experimental Psychology,**20*(3), 241–248. 10.1080/14640746808400158 5683763 10.1080/14640746808400158

[CR80] Rajan, R. & Cainer, K.E. (2008) Ageing without hearing loss or cognitive impairment causes a decrease in speech intelligibility only in informational maskers. *Neuroscience,**154*(2), 784–795. 10.1016/j.neuroscience.2008.03.067 18485606 10.1016/j.neuroscience.2008.03.067

[CR81] Rogers, C. L., Lister, J. J., Febo, D. M., Besing, J. M., & Abrams, H. B. (2006). Effects of bilingualism, noise, and reverberation on speech perception by listeners with normal hearing. *Applied Psycholinguistics,**27*(3), 465–485. 10.1017/S014271640606036X

[CR82] Rönnberg, J., Lunner, T., Zekveld, A., Sörqvist, P., Danielsson, H., Lyxell, B., & Rudner, M. (2013). The ease of language understanding (ELU) model: Theoretical, empirical, and clinical advances. *Frontiers in Systems Neuroscience,**7*, 31. 10.3389/fnsys.2013.00031 23874273 10.3389/fnsys.2013.00031 PMC3710434

[CR83] Ryherd, E., Okcu, S., Hsu, T., & Mahapatra, A. (2011). Hospital noise and occupant response. *ASHRAE Transactions,**117*(1), 248.

[CR84] Salthouse, T. A. (2019). Trajectories of normal cognitive aging. *Psychology and Aging,**34*(1), 17–24. 10.1037/pag0000288 30211596 10.1037/pag0000288 PMC6367038

[CR85] Schafer, E. C., Pogue, J., & Milrany, T. (2012). List equivalency of the AzBio sentence test in noise for listeners with normal-hearing sensitivity or cochlear implants. *Journal of the American Academy of Audiology,**23*(07), 501–509. 10.3766/jaaa.23.7.2 22992257 10.3766/jaaa.23.7.2

[CR86] Schoof, T., & Rosen, S. (2014). The role of auditory and cognitive factors in understanding speech in noise by normal-hearing older listeners. *Frontiers in Aging Neuroscience,**6*, 307. 10.3389/fnagi.2014.00307 25429266 10.3389/fnagi.2014.00307 PMC4228854

[CR87] Shen, J., & Wu, J. (2022). Speech recognition in noise performance measured remotely versus in-laboratory from older and younger listeners. *Journal of Speech, Language, and Hearing Research.*10.1044/2022_JSLHR-21-00557 10.1044/2022_JSLHR-21-00557 PMC9567433 35442717

[CR88] Shukla, A., Nieman, C. L., Price, C., Harper, M., Lin, F. R., & Reed, N. S. (2019). Impact of hearing loss on patient-provider communication among hospitalized patients: A systematic review. *American Journal of Medical Quality,**34*(3), 284–292. 10.1177/1062860618798926 30196712 10.1177/1062860618798926

[CR89] Shukla, B., Rao, B. S., Saxena, U., & Verma, H. (2018). Measurement of speech in noise abilities in laboratory and real-world noise. *Indian Journal of Otology,**24*(2), 109. 10.4103/indianjotol.INDIANJOTOL_134_17

[CR90] Sommers, M. S., & Danielson, S. M. (1999). Inhibitory processes and spoken word recognition in young and older adults: The interaction of lexical competition and semantic context. *Psychology and Aging,**14*(3), 458–472. 10.1037/0882-7974.14.3.458 10509700 10.1037//0882-7974.14.3.458

[CR91] Stuart, A., & Phillips, D. P. (1996). Word recognition in continuous and interrupted broadband noise by young normal-hearing, older normal-hearing, and presbyacusic listeners. *Ear and Hearing,**17*(6), 478–489. 8979036 10.1097/00003446-199612000-00004

[CR92] Sun, R., Karaca, Z., & Wong, H. S. (2018). *Trends in Hospital Inpatient Stays by Age and Payer, 2000–2015*. https://www.hcup-us.ahrq.gov/reports/statbriefs/sb235-Inpatient-Stays-Age-Payer-Trends.jsp 29578670

[CR93] Tainter, C. R., Levine, A. R., Quraishi, S. A., Butterly, A. D., Stahl, D. L., Eikermann, M., Kaafarani, H. M., & Lee, J. (2016). Noise levels in surgical ICUs are consistently above recommended standards. *Critical Care Medicine,**44*(1), 147–152. 26457750 10.1097/CCM.0000000000001378

[CR94] Taler, V., Aaron, G. P., Steinmetz, L. G., & Pisoni, D. B. (2010). Lexical neighborhood density effects on spoken word recognition and production in healthy aging. *Journals of Gerontology Series B: Psychological Sciences and Social Sciences,**65*(5), 551–560. 10.1093/geronb/gbq039 20542997 10.1093/geronb/gbq039 PMC2920945

[CR95] Tun, P. A., O’Kane, G., & Wingfield, A. (2002). Distraction by competing speech in young and older adult listeners. *Psychology and Aging,**17*(3), 453–467. 10.1037/0882-7974.17.3.453 12243387 10.1037//0882-7974.17.3.453

[CR96] Tun, P. A., & Wingfield, A. (1999). One voice too many: Adult age differences in language processing with different types of distracting sounds. *The Journals of Gerontology: Series B,**54B*(5), P317–P327. 10.1093/geronb/54B.5.P317 10.1093/geronb/54b.5.p317 10542824

[CR97] van Heuven, W. J., Mandera, P., Keuleers, E., & Brysbaert, M. (2014). SUBTLEX-UK: A new and improved word frequency database for British English. *Quarterly Journal of Experimental Psychology,**67*(6), 1176–1190. 10.1080/17470218.2013.850521 10.1080/17470218.2013.850521 24417251

[CR98] Verhaeghen, P. (2003). Aging and vocabulary scores: A meta-analysis. *Psychology and Aging,**18*(2), 332–339. 10.1037/0882-7974.18.2.332 12825780 10.1037/0882-7974.18.2.332

[CR99] Weinstein, N. D. (1982). Community noise problems: Evidence against adaptation. *Journal of Environmental Psychology,**2*(2), 87–97. 10.1016/S0272-4944(82)80041-8

[CR100] Wickham, H. (2016). Programming with ggplot2. Ggplot2: elegant graphics for data analysis, 241–253

[CR101] Wong, L. L. N., Ng, E. H. N., & Soli, S. D. (2012). Characterization of speech understanding in various types of noise. *The Journal of the Acoustical Society of America,**132*(4), 2642–2651. 10.1121/1.4751538 23039457 10.1121/1.4751538

[CR102] Woods, K. J. P., Siegel, M. H., Traer, J., & McDermott, J. H. (2017). Headphone screening to facilitate web-based auditory experiments. *Attention, Perception, and Psychophysics,**79*(7), 2064–2072. 10.3758/s13414-017-1361-2 10.3758/s13414-017-1361-2 PMC5693749 28695541

[CR103] Yoon, C., Feinberg, F., Luo, T., Hedden, T., Gutchess, A. H., Chen, H.-Y.M., Mikels, J. A., Jiao, S., & Park, D. C. (2004). A cross-culturally standardized set of pictures for younger and older adults: American and Chinese norms for name agreement, concept agreement, and familiarity. *Behavior Research Methods, Instruments, and Computers,**36*(4), 639–649. 10.3758/BF03206545 15641410 10.3758/bf03206545

[CR104] Yoshida, Y., & Yoshida, Y. (2014). Patient’s recognition level of medical terms as estimated by pharmacists. *Environmental Health and Preventive Medicine,**19*(6), 414–421. 10.1007/s12199-014-0408-4 25182140 10.1007/s12199-014-0408-4 PMC4235856

[CR105] Zelinski, E. M., & Hyde, J. C. (1996). Old words, new meanings: Aging and sense creation. *Journal of Memory and Language,**35*(5), 689–707. 10.1006/jmla.1996.0036

